# Supplementary material for: Understanding the complexity of socioeconomic disparities in smoking prevalence in Sweden: a cross-sectional study applying intersectionality theory
Source: BMJ Open. 2021 Feb 11;11(2):e042323. doi: 10.1136/bmjopen-2020-042323 (PMC7880088; doi:10.1136/bmjopen-2020-042323)
Supplement: Supplementary data [file bmjopen-2020-042323supp001.pdf]

Supplementary material 1

S1.

Trends in smoking prevalence in the National Health Surveys for Sweden. Categories of age, gender, educational achievement, migration status and household composition are shown with different lines and 95% confidence intervals in the respective graphs.

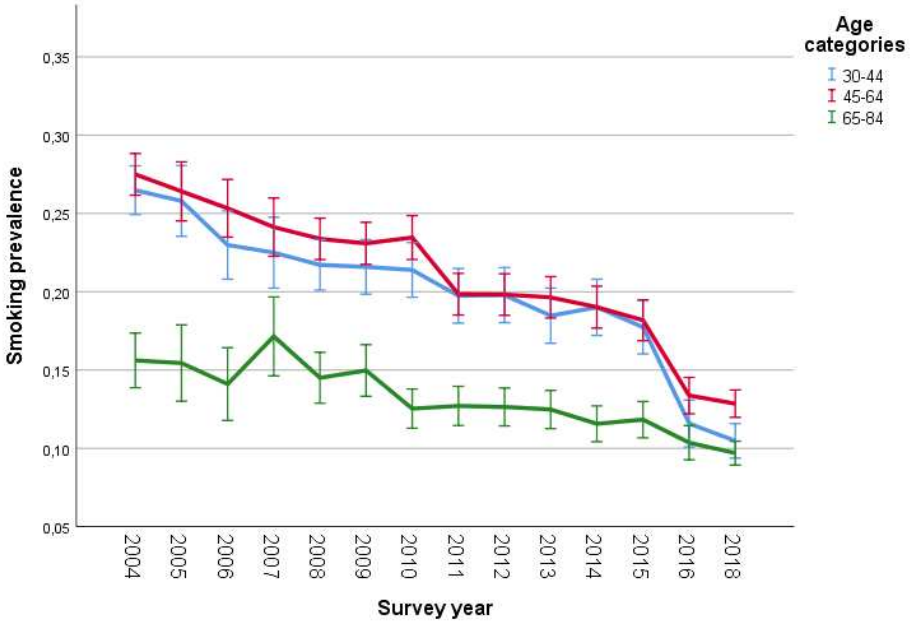

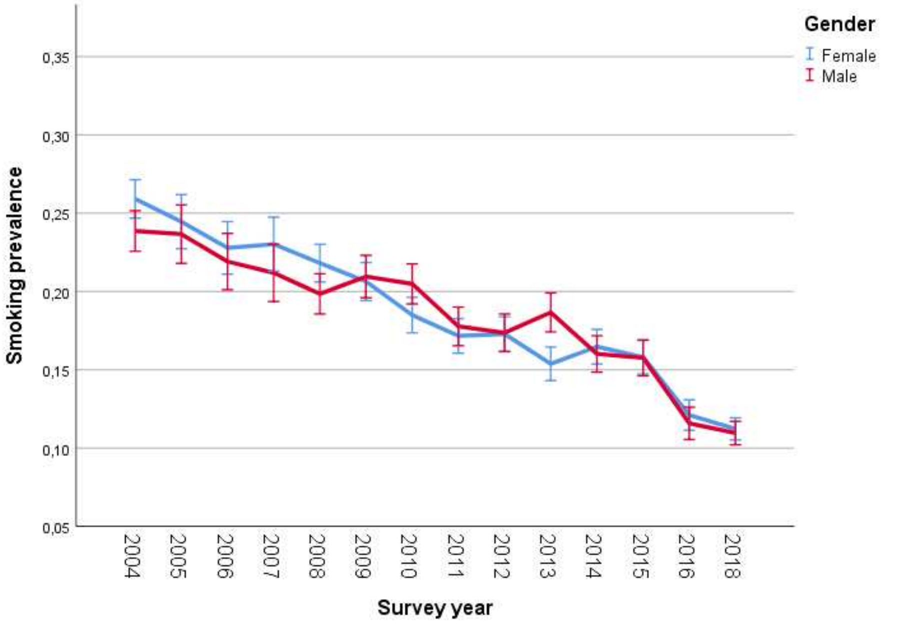

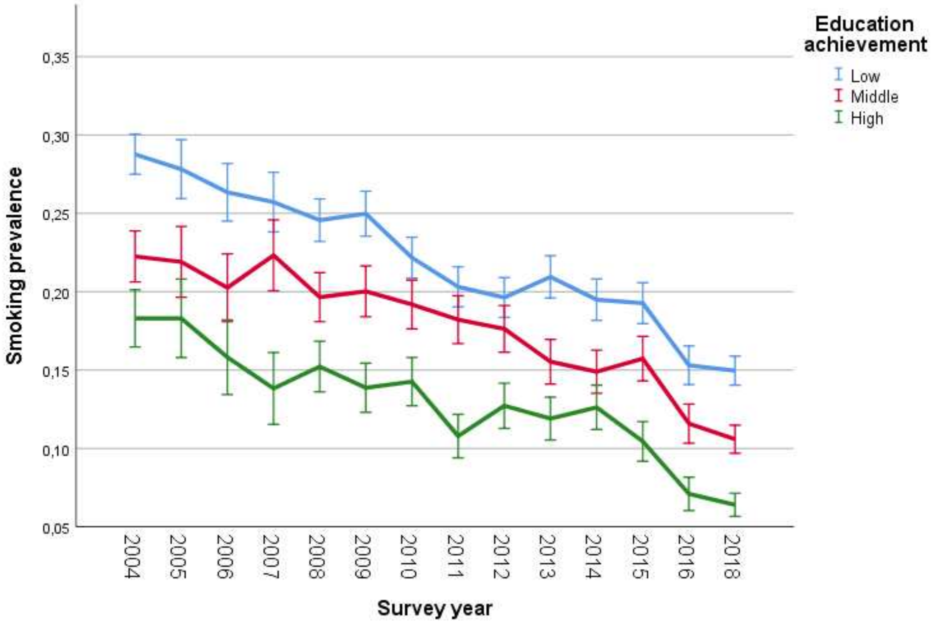
Education achievement
Low
Middle
High
Smoking prevalence
0,35
0,30
0,25
0,20
0,15
0,10
0,05
2004
2005
2006
2007
2008
2009
2010
2011
2012
2013
2014
2015
2016
2018
Survey year

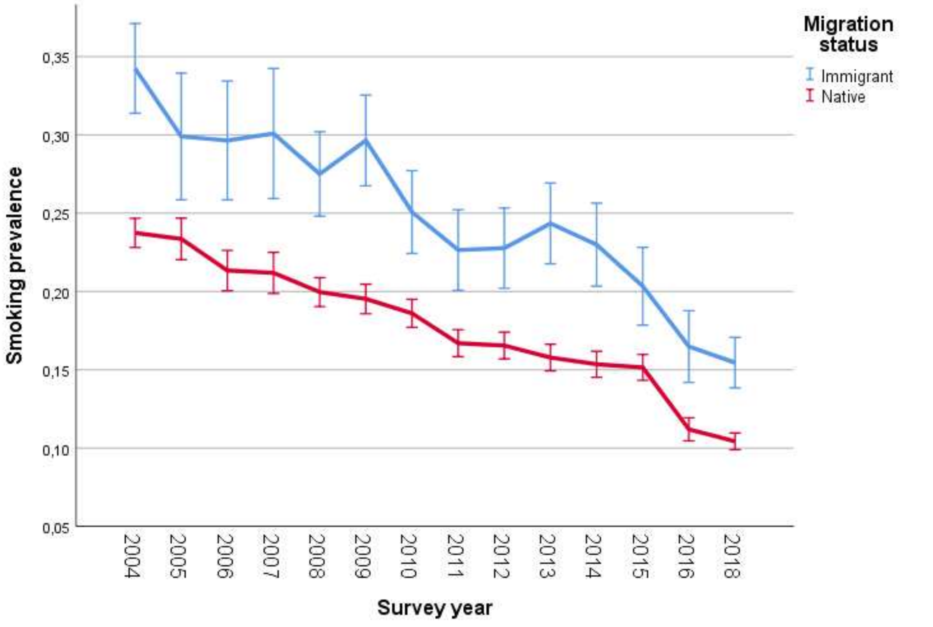
Migration status
Immigrant
Native
Smoking prevalence
0,35
0,30
0,25
0,20
0,15
0,10
0,05
2004
2005
2006
2007
2008
2009
2010
2011
2012
2013
2014
2015
2016
2018
Survey year

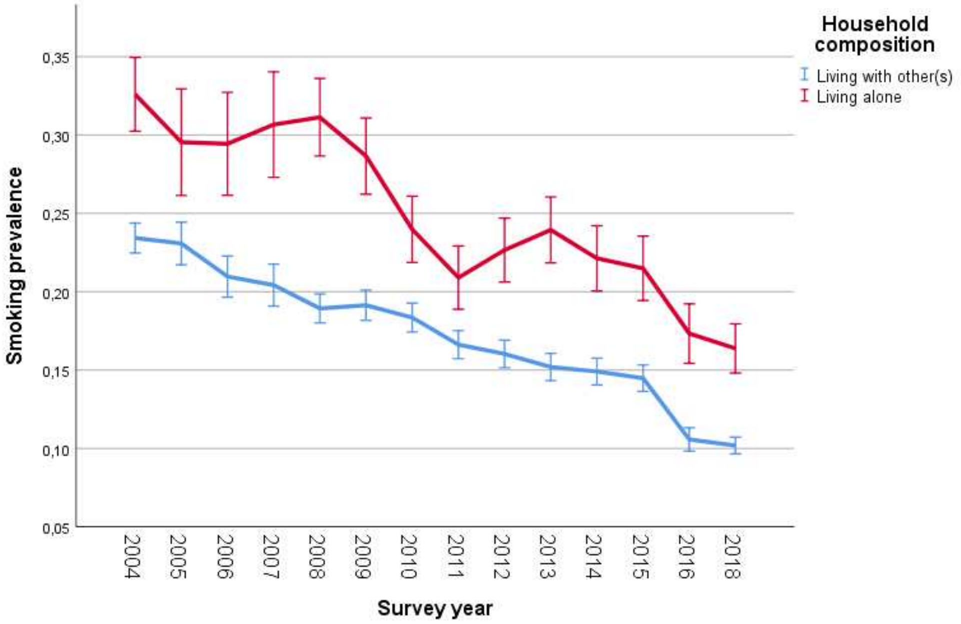
Household composition
Living with other(s)
Living alone
Smoking prevalence
0,35
0,30
0,25
0,20
0,15
0,10
0,05
2004
2005
2006
2007
2008
2009
2010
2011
2012
2013
2014
2015
2016
2018
Survey year

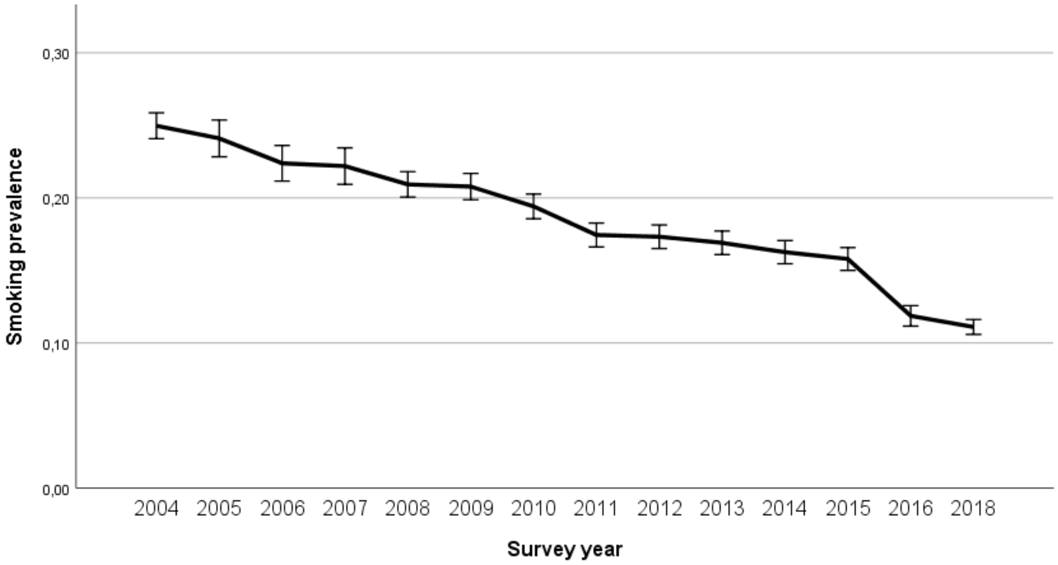
Smoking prevalence
0,30
0,20
0,10
0,00
2004
2005
2006
2007
2008
2009
2010
2011
2012
2013
2014
2015
2016
2018
Survey year
