# Supplementary material for: Understanding the complexity of socioeconomic disparities in smoking prevalence in Sweden: a cross-sectional study applying intersectionality theory
Source: BMJ Open. 2021 Feb 11;11(2):e042323. doi: 10.1136/bmjopen-2020-042323 (PMC7880088; doi:10.1136/bmjopen-2020-042323)
Supplement: Supplementary data [file bmjopen-2020-042323supp002.pdf]

Supplementary material 2

S2

Table 2, full version. Results from the intersectional model 7 indicating the Prevalence Ratios (PR) with 95% confidence intervals (CI) of smoking across intersectional strata in the Swedish population using the stratum of young, native, men with high education that were living with other(s) (LWO) as reference in the comparisons.

Age	Gender	Educational achievement	Migration status	Household composition	PR (95% CI)
30-44	Female	Low	Immigrant	LWO	2.35 (1.96-2.82)
30-44	Female	Low	Immigrant	Living alone	3.41 (1.96-5.94)
30-44	Female	Low	Native	LWO	2.24 (1.96-2.56)
30-44	Female	Low	Native	Living alone	2.95 (2.29-3.78)
30-44	Female	Middle	Immigrant	LWO	1.83 (1.51-2.21)
30-44	Female	Middle	Immigrant	Living alone	2.33 (1.34-4.05)
30-44	Female	Middle	Native	LWO	1.53 (1.35-1.73)
30-44	Female	Middle	Native	Living alone	2.2 (1.8-2.7)
30-44	Female	High	Immigrant	LWO	1.22 (0.99-1.49)
30-44	Female	High	Immigrant	Living alone	2.87 (1.86-4.42)
30-44	Female	High	Native	LWO	0.86 (0.74-0.98)
30-44	Female	High	Native	Living alone	1.72 (1.39-2.12)
30-44	Male	Low	Immigrant	LWO	3.66 (3.07-4.35)
30-44	Male	Low	Immigrant	Living alone	4.45 (3.29-6.03)
30-44	Male	Low	Native	LWO	1.92 (1.68-2.2)
30-44	Male	Low	Native	Living alone	2.67 (2.21-3.21)
30-44	Male	Middle	Immigrant	LWO	2.84 (2.36-3.43)
30-44	Male	Middle	Immigrant	Living alone	3.33 (2.35-4.71)
30-44	Male	Middle	Native	LWO	1.43 (1.25-1.63)
30-44	Male	Middle	Native	Living alone	2.21 (1.85-2.64)
30-44	Male	High	Immigrant	LWO	2.13 (1.74-2.6)
30-44	Male	High	Immigrant	Living alone	2.32 (1.53-3.5)
30-44	Male	High	Native	LWO	Reference

30-44	Male	High	Native	Living alone	1.75 (1.41-2.18)
45-64	Female	Low	Immigrant	LWO	2.19 (1.88-2.55)
45-64	Female	Low	Immigrant	Living alone	3.22 (2.56-4.06)
45-64	Female	Low	Native	LWO	2.08 (1.85-2.34)
45-64	Female	Low	Native	Living alone	2.99 (2.61-3.41)
45-64	Female	Middle	Immigrant	LWO	1.87 (1.56-2.23)
45-64	Female	Middle	Immigrant	Living alone	2.23 (1.66-3.01)
45-64	Female	Middle	Native	LWO	1.29 (1.14-1.46)
45-64	Female	Middle	Native	Living alone	2.16 (1.84-2.53)
45-64	Female	High	Immigrant	LWO	1.27 (1.03-1.57)
45-64	Female	High	Immigrant	Living alone	1.63 (1.08-2.45)
45-64	Female	High	Native	LWO	0.97 (0.84-1.11)
45-64	Female	High	Native	Living alone	1.76 (1.46-2.13)
45-64	Male	Low	Immigrant	LWO	2.55 (2.18-2.98)
45-64	Male	Low	Immigrant	Living alone	3.61 (2.9-4.5)
45-64	Male	Low	Native	LWO	1.71 (1.52-1.93)
45-64	Male	Low	Native	Living alone	2.77 (2.42-3.17)
45-64	Male	Middle	Immigrant	LWO	2.39 (2.01-2.86)
45-64	Male	Middle	Immigrant	Living alone	3.1 (2.26-4.26)
45-64	Male	Middle	Native	LWO	1.28 (1.12-1.45)
45-64	Male	Middle	Native	Living alone	1.92 (1.61-2.31)
45-64	Male	High	Immigrant	LWO	1.91 (1.56-2.35)
45-64	Male	High	Immigrant	Living alone	2.7 (1.84-3.98)
45-64	Male	High	Native	LWO	0.92 (0.8-1.07)
45-64	Male	High	Native	Living alone	1.35 (1.04-1.75)
65-84	Female	Low	Immigrant	LWO	1.23 (0.98-1.54)
65-84	Female	Low	Immigrant	Living alone	1.62 (1.28-2.05)
65-84	Female	Low	Native	LWO	1.1 (0.97-1.25)
65-84	Female	Low	Native	Living alone	1.62 (1.42-1.86)

65-84	Female	Middle	Immigrant	LWO	1.18 (0.81-1.71)
65-84	Female	Middle	Immigrant	Living alone	1.66 (1.12-2.46)
65-84	Female	Middle	Native	LWO	0.8 (0.66-0.96)
65-84	Female	Middle	Native	Living alone	1.2 (0.97-1.48)
65-84	Female	High	Immigrant	LWO	0.61 (0.33-1.11)
65-84	Female	High	Immigrant	Living alone	1.16 (0.65-2.07)
65-84	Female	High	Native	LWO	0.55 (0.45-0.69)
65-84	Female	High	Native	Living alone	0.83 (0.64-1.06)
65-84	Male	Low	Immigrant	LWO	1.57 (1.27-1.95)
65-84	Male	Low	Immigrant	Living alone	2.49 (1.84-3.37)
65-84	Male	Low	Native	LWO	0.96 (0.84-1.1)
65-84	Male	Low	Native	Living alone	1.71 (1.47-2)
65-84	Male	Middle	Immigrant	LWO	1.12 (0.82-1.51)
65-84	Male	Middle	Immigrant	Living alone	1.29 (0.74-2.24)
65-84	Male	Middle	Native	LWO	0.85 (0.73-0.99)
65-84	Male	Middle	Native	Living alone	1.47 (1.18-1.83)
65-84	Male	High	Immigrant	LWO	1.06 (0.72-1.57)
65-84	Male	High	Immigrant	Living alone	0.91 (0.38-2.21)
65-84	Male	High	Native	LWO	0.58 (0.48-0.71)
65-84	Male	High	Native	Living alone	1.19 (0.88-1.6)
