# Supplementary material for: Understanding the complexity of socioeconomic disparities in smoking prevalence in Sweden: a cross-sectional study applying intersectionality theory
Source: BMJ Open. 2021 Feb 11;11(2):e042323. doi: 10.1136/bmjopen-2020-042323 (PMC7880088; doi:10.1136/bmjopen-2020-042323)
Supplement: Supplementary data [file bmjopen-2020-042323supp003.pdf]

Supplementary material 3

S3

Sensitivity analysis where income tertiles were used as indicators of socioeconomic position instead of educational achievement.

Table 1. Results from the intersectional model 7 indicating the 10 highest and 10 lowest Prevalence Ratio (PR) with 95% confidence intervals (CI) of smoking across intersectional strata in the Swedish population using the stratum of young, native, men with high income that were living with other(s) (LWO) as reference in the comparisons.

Age	Gender	Income	Migration status	Civil status	PR (95% CI)
65-84	Male	High	Immigrant	LWO	0.51 (0.07-3.61)
65-84	Female	High	Immigrant	Living alone	0.64 (0.37-1.08)
65-84	Male	High	Native	Living alone	0.64 (0.55-0.74)
65-84	Female	High	Native	Living alone	0.66 (0.56-0.78)
65-84	Female	High	Native	LWO	0.71 (0.45-1.12)
65-84	Male	Middle	Native	Living alone	0.74 (0.65-0.83)
65-84	Female	Middle	Native	Living alone	0.76 (0.67-0.86)
65-84	Male	High	Immigrant	Living alone	0.77 (0.51-1.15)
65-84	Male	Low	Native	Living alone	0.87 (0.76-0.99)
65-84	Male	High	Native	LWO	0.91 (0.57-1.46)
30-44	**Male**	**High**	**Native**	**LWO**	**Reference**
30-44	Male	High	Immigrant	Living alone	2.37 (0.98-5.73)
45-64	Male	Low	Immigrant	LWO	2.53 (2.14-2.98)
65-84	Female	High	Immigrant	Living alone	2.58 (0.96-6.89)
30-44	Female	High	Immigrant	Living alone	2.62 (0.98-7.02)
45-64	Male	Middle	Immigrant	Living alone	2.63 (1.86-3.7)
30-44	Female	Low	Immigrant	Living alone	2.64 (1.89-3.68)
30-44	Male	Middle	Immigrant	Living alone	2.66 (1.74-4.08)
30-44	Male	Low	Immigrant	LWO	2.73 (2.32-3.2)
30-44	Male	Low	Immigrant	Living alone	2.94 (2.31-3.74)
45-64	Male	Low	Immigrant	Living alone	2.96 (2.45-3.58)
AUC					0.65
ΔAUC compared with model 6					0.01

Table 2. Prevalence ratios (PR) and 95%-confidence intervals (CI), of smoking among people aged 30-84 included in the National Health Surveys between 2004 and 2018 in relation to survey year, age, gender, income, migration- and household composition. Model 7 includes the same variables as model 6 but as a multicategorical variable, The PRs for model 7 are presented in the table 1. AUC-values with 95%CI representing the discriminatory accuracy and ΔAUC-values of the models are also presented.

		Model 1	Model 2	Model 3	Model 4	Model 5	Model 6
Year							
	2004	2.25 (2.11-2.40)	2.08 (1.95-2.21)	2.08 (1.95-2.21)	1.85 (1.74.1.98)	1.88 (1.76-2.00)	1.84 (1.73-1.97)
	2005	2.17 (2.01-2.35)	2.01 (1.86.1.17)	2.01 (1.95-2.21)	1.83 (1.69-1.98)	1.85 (1.71-2.00)	1.83 (1.69-1.98)
	2006	2.02 (1.86-2.18)	1.87 (1.72-2.02)	1.87 (1.72-2.02)	1.70 (1.57-1.84)	1.71 (1.58-1.85)	1.68 (1.55-1.82)
	2007	2.00 (1.84-2.17)	1.86 (1.71-2.01)	1.86 (1.71-2.01)	1.71 (1.57-1.85)	1.73 (1.59-1.87)	1.70 (1.56-1.84)
	2008	1.89 (1.76-2.02)	1.76 (1.64-1.88)	1.76 (1.64-1.88)	1.64 (1.53-1.75)	1.65 (1.54-1.77)	1.63 (1.52-1.74)
	2009	1.87 (1.75-2.01)	1.75 (1.64.1.88)	1.76 (1.64-1.88)	1.65 (1.54-1.77)	1.66 (1.55-1.78)	1.63 (1.52-1.75)
	2010	1.75 (1.63-1.87)	1.70 (1.58-1.82)	1.70 (1.58-1.82)	1.61 (1.50-1.72)	1.62 (1.51-1.73)	1.59 (1.48-1.70)
	2011	1.57 (1.46-1.69)	1.53 (1.43-1.64)	1.53 (1.43-1.64)	1.45 (1.35.1.56)	1.46 (1.36-1.57)	1.43 (1.33-1.54)
	2012	1.56 (1.45-1.68)	1.53 (1.42-1.64)	1.53 (1.42-1.64)	1.47 (1.37-1.57)	1.47 (1.37-1.58)	1.44 (1.34-1.55)
	2013	1.52 (1.42-1.64)	1.49 (1.39-1.60)	1.49 (1.39-1.60)	1.45 (5 (1.35.1,55)	1.45 (1.35-1.56)	1.42 (1.32-1.52)
	2014	1.46 (1.36-1.57)	1.45 (1.35-1.56)	1.45 (1.35-1.56)	1.41 (1.31-1.52)	1.42 (1.32-1.53)	1.39 (1.30-1.50)
	2015	1.42 (1.32-1.53)	1.40 (1.30-1.51)	1.40 (1.30-1.51)	1.37 (1.27-1.47)	1.38 (1.28-1.48)	1.35 (1.26-1.46)
	2016	1.07 (0.99-1.16)	1.06 (0.97-1.14)	1.06 (0.97-1.14)	1.04 (0.96-1.13)	1.05 (0.97-1.13)	1.03 (0.95-1.11)
	2018	Reference	Reference	Reference	Reference	Reference	Reference
Age							
	30-44		Reference	Reference	Reference	Reference	Reference
	45-64		1.06 (1.03-1.10)	1.06 (1.03-1.10)	0.94 (0.91-0.97)	0.94 (0.91-0.98)	0.93 (0.90-0.96)
	65-84		068 (0.65-0.71)	0.68 (1.86-0.71)	0.56 (0.53-0.58)	0.57 (0.55-0.59)	0.53 (0.51.0.56)
Gender							
	Male			Reference	Reference	Reference	Reference
	Female			1.00 (0.97-1.02)	1.02 (0.99-1.05)	1.02 (0.99-1.05)	1.01 (0.98-1.04)
Income							
	Low				1.96 (1.88-2.04)	1.96 (1.88-2.04)	1.93 (1.86-2.01)
	Middle				1.42 (1.36-1.48)	1.43 (1.37-1.45)	1.42 (1.36-1.49)
	High				Reference	Reference	Reference

Born in Sweden							
	Native					Reference	Reference
	Immigrant					1.39 (1.34-1.45)	1.39 (1.34-1.44)
Household composition							
	Living alone						1.53 (1.48-1.58)
	Living with other(s)						Reference
AUC		0.58 (0.58-0.59)	0.60 (0.60-0.61)	0.60 (0.60-0.61)	0.64 (0.63-0.64)	0.64 (0.64-0.64)	0.64 (0.64-0.65)
ΔAUC		-	0.02	0.00	0.04	0.00	0.01
