# Supplementary material for: Understanding the complexity of socioeconomic disparities in smoking prevalence in Sweden: a cross-sectional study applying intersectionality theory
Source: BMJ Open. 2021 Feb 11;11(2):e042323. doi: 10.1136/bmjopen-2020-042323 (PMC7880088; doi:10.1136/bmjopen-2020-042323)
Supplement: Supplementary data [file bmjopen-2020-042323supp004.pdf]

Supplementary material 4

S4. Smoking prevalence across regions of birth among 110 044 individuals responding National Health Surveys during 2004 – 2016 and 2018. Both everyday smokers and sometimes smokers are included in the proportions presented.

Region of birth	Women	Men
Sweden	17.5%	16.4%
Nordic countries	21.7%	23.0%
Europe	24.0%	27.1%
Outside Europe	16.3%	32.5%
