# Supplementary material for: Understanding the complexity of socioeconomic disparities in smoking prevalence in Sweden: a cross-sectional study applying intersectionality theory
Source: BMJ Open. 2021 Feb 11;11(2):e042323. doi: 10.1136/bmjopen-2020-042323 (PMC7880088; doi:10.1136/bmjopen-2020-042323)
Supplement: Author's manuscript [file bmjopen-2020-042323.draft_revisions.pdf]

BMJ Open is committed to open peer review. As part of this commitment we make the peer review history of every article we publish publicly available.

When an article is published we post the peer reviewers' comments and the authors' responses online. We also post the versions of the paper that were used during peer review. These are the versions that the peer review comments apply to.

The versions of the paper that follow are the versions that were submitted during the peer review process. They are not the versions of record or the final published versions. They should not be cited or distributed as the published version of this manuscript.

BMJ Open is an open access journal and the full, final, typeset and author-corrected version of record of the manuscript is available on our site with no access controls, subscription charges or pay-per-view fees (http://bmjopen.bmj.com).

If you have any questions on BMJ Open's open peer review process please email info.bmjopen@bmj.com

BMJ Open

Understanding the Complexity of Socioeconomic Disparities in smoking prevalence: Combining Intersectionality theory with analysis of individual heterogeneity and discriminatory accuracy (AIHDA)

Journal:	*BMJ Open*
Manuscript ID	bmjopen-2020-042323
Article Type:	Original research
Date Submitted by the Author:	05-Jul-2020

Complete List of Authors:	Axelsson Fisk, Sten; Lund University, Unit for Social Epidemiology, Department of Clinical Sciences<br>Lindstrom, Martin; Lunds University Faculty of Medicine, Unit for Social Medicine and Health Policy, Department of Clinical Sciences; Region Skåne, Centre for Primary Health Care Research<br>Perez-Vicente, Raquel; Lunds University Faculty of Medicine, Unit for Social Epidemiology, Department of Clinical Sciences<br>Merlo, Juan; Lunds University Faculty of Medicine, Unit for Social Epidemiology, Department of Clinical Sciences; Region Skåne, Malmö, Centre for Primary Health Care Research

Keywords:	SOCIAL MEDICINE, EPIDEMIOLOGY, Health policy < HEALTH SERVICES ADMINISTRATION & MANAGEMENT, PUBLIC HEALTH, STATISTICS & RESEARCH METHODS, Substance misuse < PSYCHIATRY

SCHOLARONE™
Manuscripts

I, the Submitting Author has the right to grant and does grant on behalf of all authors of the Work (as defined in the below author licence), an exclusive licence and/or a non-exclusive licence for contributions from authors who are: i) UK Crown employees; ii) where BMJ has agreed a CC-BY licence shall apply, and/or iii) in accordance with the terms applicable for US Federal Government officers or employees acting as part of their official duties; on a worldwide, perpetual, irrevocable, royalty-free basis to BMJ Publishing Group Ltd ("BMJ") its licensees and where the relevant Journal is co-owned by BMJ to the co-owners of the Journal, to publish the Work in this journal and any other BMJ products and to exploit all rights, as set out in our licence.

The Submitting Author accepts and understands that any supply made under these terms is made by BMJ to the Submitting Author unless you are acting as an employee on behalf of your employer or a postgraduate student of an affiliated institution which is paying any applicable article publishing charge ("APC") for Open Access articles. Where the Submitting Author wishes to make the Work available on an Open Access basis (and intends to pay the relevant APC), the terms of reuse of such Open Access shall be governed by a Creative Commons licence – details of these licences and which Creative Commons licence will apply to this Work are set out in our licence referred to above.

Other than as permitted in any relevant BMJ Author's Self Archiving Policies, I confirm this Work has not been accepted for publication elsewhere, is not being considered for publication elsewhere and does not duplicate material already published. I confirm all authors consent to publication of this Work and authorise the granting of this licence.

For peer review only

Understanding the Complexity of Socioeconomic Disparities in smoking prevalence: Combining Intersectionality theory with analysis of individual heterogeneity and discriminatory accuracy (AIHDA)

Running Title: Complex Disparities in smoking prevalence

Journal BMJ-Open

Sten Axelsson Fisk, MD[1]
Martin Lindström MD, PhD, Prof[2,3]
Raquel Perez-Vicente. MSc[1]
Juan Merlo, MD, PhD, Prof[1,3]

[1] Unit for Social Epidemiology, Department of Clinical Sciences, Lund University, Malmö, Sweden
[2] Unit for Social Medicine and Health Policy, Department of Clinical Sciences, Lund University, Malmö, Sweden
[3] Centre for Primary Health Care Research, Region Skåne, Malmö, Sweden

Corresponding author
Sten Axelsson Fisk
Unit for Social Epidemiology, Lund University
Jan Waldenströms gata 35
214 28 Malmö
Sweden
Phone: +46-701-732506
Email: sten.axelsson_fisk@med.lu.se

Words: 3806 (excluding abstract, article summary, abbreviations and references)
Tables: 3
Figures: 2
Supplemental material: 2

For peer review only

Abstract (250 words)

Objectives: Socioeconomic disparities in smoking prevalence remains a challenge to public health. The objective of this study was to present a simple methodology that displays intersectional patterns of smoking and quantify heterogeneities within groups to avoid inappropriate and potentially stigmatizing conclusions exclusively based on group averages.

Setting: This is a cross-sectional observational study based on data from the National Health Surveys for Sweden (2004-2016 and 2018) including 136 301 individuals. We excluded people under 30 years of age, or missing information on education, cohabitation or smoking habits. The final sample consisted on 110 044 individuals or 81.4% of the original sample.

Outcome: Applying intersectional analysis of individual heterogeneity and discriminatory accuracy (AIHDA), we investigated the risk of self-reported smoking across 72 intersectional strata defined by age, gender, educational achievement, civil and migration status.

Results: The distribution of smoking habit risk in the population was very heterogeneous. For instance, immigrant men aged 30–44 that lived alone and had low educational achievement had a prevalence of smoking of 54% (95%CI 44–64%), around 9 times higher than native cohabiting women aged 65–84 with high educational achievement that had a prevalence of 6% (95%CI 5–7%). The discriminatory accuracy of the information was moderate.

Conclusion: A more detailed, intersectional mapping of the socioeconomic and demographic disparities of smoking can assist in public health management aiming to eliminate this unhealthy habit from the community. Intersectionality theory together with AIHDA provides information that can guide resource allocation according to the concept proportionate universalism.

For peer review only

Strengths and limitations

- We present an intersectional approach to study the multidimensional socioeconomic disparities in smoking prevalence in Sweden.
- In addition to differences between averages of intersectional strata, we quantify individual heterogeneities around those averages by presenting measurements of discriminatory accuracy.
- Our method is simpler but share crucial advantages with Multilevel AIHDA, such as improved health mapping and assessment of intersectional interaction.
- We use pooled data from Swedish National Health Survey with participation rates spanning from 60.8% 2004 to 42.1% 2018.
- Analysis of Individual Heterogeneity and Discriminatory Accuracy (AIHDA) is a suitable tool to inform whether interventions to reduce socioeconomic health disparities should be universal or target specific groups.

For peer review only

Abbreviations

AIHDA=Analysis of Individual Heterogeneity and Discriminatory Accuracy, AUC=Area Under receiving operator characteristics Curve, CI=Confidence Interval, DA=Discriminatory Accuracy, OR=Odds Ratio, PR=Prevalence Ratio, RR=Relative Risk

For peer review only

Introduction

A higher prevalence of smoking among individuals with low socioeconomic position (SEP) compared to higher SEP has been reported in several studies in Sweden [1] and globally [2-5]. The higher prevalence results both from higher rates of initiation [6] and lower rates of successful smoking cessation [7]. In addition to this, other factors like country of birth [8], civil status [9] as well as the age cohort and gender of an individual influence the probability of smoking [10]. Overall the socioeconomic determinants of smoking are multidimensional but few studies until now have empirically confronted this heterogeneity using an intersectional perspective [11-15].

Intersectionality theory, proportionate universalism, and the analysis of individual heterogeneity and discriminatory accuracy (AIHDA)

Launching smoking cessation campaigns is a logical step to reduce socioeconomic differences in smoking prevalence, but the poor evidence that exist [16] suggest that while such interventions may be efficient when it comes to reducing overall smoking prevalence, they seem rather inefficient when it comes to reducing socioeconomic disparities in smoking [17]. Marmot and Bell claim [18] that interventions to reduce socioeconomic health disparities need to address all levels of society and not only those who are worst off. They argue that an efficient approach may be *proportionate universalism* [18, 19] where interventions are universal, i.e. directed towards the whole population (such as tobacco taxes, smoking bans in public) but proportionately more intense among population subgroups with augmented needs where targeted interventions can be launched (i.e. information campaigns in specific neighbourhoods or populations such as pregnant women). However, as argued elsewhere [19-21] successful and efficient implementation of *proportionate universalism* requires development and application of appropriate theories and epidemiologic methodologies.

For peer review only

Intersectionality theory, originating in gender and anti-colonial research [22] is a critical social theory that stresses the need for simultaneous consideration of different social dimensions such as racialized identity, gender and class in order to properly understand the social context acting on individuals. According to intersectionality theory, the social reality is shaped by overlapping systems of oppression that influence distribution of resources and power in society.

The inclusion of intersectionality in epidemiology and public health has been promoted by several scholars [23-26]. A direct consequence of this approach in quantitative analyses is the study of multiple intersectional strata defined by combinations of different social dimensions, since the effect of each social dimension on an individual is intrinsically dependent on the other social identities of that person. This contrasts with the common approach considering one social dimension at the time. Thereby, the intersectional approach may enrich public health research by providing an improved mapping of socioeconomic health disparities. Such socioeconomic heterogeneity can be analyzed by quantifying differences between intersectional strata averages. However, we [20, 25-28] and other scholars [29-31] stress the added relevance of simultaneously quantifying the discriminatory accuracy (DA) of the intersectional categorization for specific outcomes. An intersectional map combined with information on its DA provides an improved picture of the socioeconomic heterogeneity existing in the society. This approach can be used to inform interventions according to the concept of proportionate universalism, where universal measures are paired with actions directed to specific intersectional strata in proportion to their level of risk [18]. The extent to which a universal intervention needs to be proportional can be evaluated by the DA of the

For peer review only

intersectional strata. A low DA suggests the need for universal interventions while a high DA support more selective interventions.

Adopting a quantitative perspective, in the present study we aim to illustrate how a more precise intersectional categorisation combined with the analysis of individual heterogeneity and DA (AIHDA) improves our understanding of socioeconomic inequalities in smoking and facilitates the application of proportionate universalism. We do so by analysing the National Health Surveys (NHS) for Sweden.

Methods

Study population

In this cross-sectional observational study, we used data from all the 14 National Health Surveys (NHS) for Sweden for the years 2004–2016 and 2018 (https://www.folkhalsomyndigheten.se/the-public-health-agency-of-sweden/public-health-reporting/). The NHS is an ongoing collaborative project between the Public Health Agency of Sweden and the Swedish Association of Local Authorities and Regions (SALAR). The NHS record self-reported information on health, lifestyle and living conditions. The study has been conducted annually between 2004 and 2016 and comprised a random sample of 20,000 individuals aged 16–84 years. After 2016 the survey is conducted biannually but with a random sample of 40,000 individuals. Response rates span from 60.8% 2004 to 42.1% 2018. Using a unique personal identification number, the Swedish authorities linked the sample surveys to national register administered at Statistics Sweden to obtain demographical and socioeconomic information.

For peer review only

For our study we pooled the data from the last 14 surveys, which rendered a sample of 136 301 individuals. Thereafter, we excluded people under 30 years of age, or missing information on education, cohabitation or smoking habits. The final sample consisted on 110 044 individuals or 81.4% of the original sample (Figure 1).

The present investigation was approved by the Swedish Ethical Review Authority (Dnr: 2019-01793) and the data safety committee at the Public Health Agency of Sweden.

Patient and public involvement

All data from NHS provided to researchers is anonymised, so study participants cannot be identified. The study participants were not involved in the research process.

Assessment of variables

Smoking status was assessed based on the answer to the question "Do you smoke?", if the person answered "Yes" or "Yes, sometimes", the individual was categorized as a smoker, if the respondent answered "No" the individual was considered a non-smoker.

We categorized *age* into three groups: 30-44, 45-64 and 65-84-year-old. We classified gender as a binary variable distinguishing between men and women as more specific information on gender was not available in the questionnaire. We classified *educational achievement* into three categories, as low if the respondent had not completed three years of high school education, as middle if they had high school education but less than three years of education after high school and high if the respondent had at least three years of education after high school. Throughout 2008-2016 respondents were asked "with whom do you share household?", we defined *civil status* as living alone if the respondent answered "with no one", otherwise as cohabiting. In 2018 that question was not asked so individuals were defined in the same way according to the information provided by Statics Sweden. We classified *migration status* as native (i.e., born in Sweden) or immigrant.

For peer review only

As a way of operationalising intersectional contexts, we created 72 strata by combining the three categories of age, the two of gender, the three of educational achievement, the two of migration status and the two categories of civil status. We used 30–45-year-old, native men cohabiting and with high educational achievement as the reference in the comparisons, as this group was assumed to occupy the position of greatest structural privilege. We also included the survey year of the participants using 2018 as reference in all comparisons.

Statistical analyses

The first step in our analysis was to obtain the trends in smoking prevalence and the trends in socioeconomic and demographic gradients in smoking between 2004 and 2018 (see supplementary information S1). Thereafter, we performed a stratified analysis aimed to provide a detailed map of the prevalence (i.e., absolute risk) and 95% confidence intervals (CI) of smoking across the intersectional strata. This stratification allows comparing the prevalence of smoking in different strata without any reference (Figure 2).

Thereafter, we performed seven consecutive regression analyses, modelling smoking as the dependent variable and survey year as well as the different demographical and socioeconomic dimensions alone and in combination as explanatory variables. The use of logistic regression to obtain odds ratios (OR) is common but the OR is a good estimation of the relative risk (RR) only when the prevalence of the outcome is very small (rare event assumption) [32]. Therefore, for the analysis, rather than logistic regression to obtain ORs, we used Cox proportional hazards regression with a constant follow-up time equal to one to obtain prevalence ratios (PR) [33] with 95% CI.

Model 1 included only survey year, model 2 added age, model 3 added gender, model 4 added educational achievement, model 5 added migration status and model 6 added civil status and thus included all the variables that defined the intersectional strata. Finally, the

For peer review only

intersectional model 7 included the same variables as model 6 but in the form of a multicategorical variable with 72 intersectional strata. Here, we used the 30–45-year-old, native men cohabiting and with high educational achievement as the reference in the comparison.

For each model, we quantified its DA by means of the area under the receiver operator characteristics curve (AUC) [34]. The AUC measures the accuracy of the information provided by the variables in the model for discriminating individuals who smoke from those who don't. The AUC takes a value between 0.5 and 1, where 1 indicates perfect discrimination and 0.5 means that the studied variables have no DA at all. The AUC can even be used to qualify the size of the intersectional differences. Rather than evaluating the absolute risk differences between strata, using the AUC we assess the overlapping of the individual risk predictions (based on the intersectional strata) between smokers and non-smokers.

There is no fully established practical guideline for the interpretation of the size of the AUC as a measure of DA when analysing intersectional inequalities. However, based on the classification provided by Hosmer and Lemeshow [35] we qualify intersectional inequalities according to the DA as (i) "absent or very small" (AUC= 0.5–0.6), (ii) "moderate" (AUC >0.6–≤ 0.7), (iii) "large" (AUC >0.7– ≤ 0.8) and (iv) "very large" (AUC >0.8). Evaluating intersectional differences using only strata prevalence is insufficient as it does not consider any overlapping between the strata. Therefore, the AUC provides fundamental information for evaluation of group differences [36].

We further calculated the incremental change in the AUC value (Δ-AUC) between the models. The Δ-AUC quantifies the improvement in the DA obtained by a model, in relation to the previous model [21]. The categorical intersectional variable in model 7 allows for the

For peer review only

capturing of interaction of effects. If any such interaction exists, the DA of model 7 will increase in comparison with model 6 and the Δ-AUC will thus be positive.

We used STATA version 15.1 and IBM SPSS (Statistical Package for the Social Sciences) version 25 for PC to perform all statistical analyses.

Results

Over the whole study period, the prevalence of smoking was 18%. The visual analysis of the trends indicated that the prevalence of smoking monotonically decreased in Sweden from 25.0% in 2004 to around 11.1% in 2018. We observed consistent differences between groups defined by age, gender, country of birth, educational achievement and civil status. However, in absolute terms, such differences were similar along the years (see supplementary information S1).

Table 1 presents the prevalence of smokers and non-smokers across the included socioeconomic and demographic variables as well as across survey years. It indicates that the prevalence of smoking was higher in individuals aged 45-64 years (20.6%) than in both younger (19.8%) and older people (12.4%). Women and men had similar prevalence of smoking (17.9% vs 17.8%). As expected, smoking was more common among people with low (21.7%) and medium (17.0%) educational achievement compared to people with high educational achievement (11.9%). The prevalence of smoking was higher among immigrants (23.9%) than among natives (17.0%) and the same was true for individuals living alone (24.1%) compared to those who were cohabiting (16.5%).

Figure 2 shows the prevalence of smoking across the intersectional strata. We observed the highest prevalence (54%) among 30-44-year-old immigrant men with low educational achievement and living alone, and the lowest prevalence (6%) among 65-84-year-old native women with high educational achievement and living together. The reference stratum (i.e.,

For peer review only

30–45-year-old, native men cohabiting and with high educational achievement) used in the relative comparisons (Table 2) presented a smoking prevalence of about 12%.

The table 3 informs that the RR of smoking decreases with age, being lowest in the old population. This age gradient is clear after adjustment for the other variables in the model 6. Low educational achievement, being immigrant and living alone was associated with a higher smoking risk. However, there were no age-adjusted gender differences. The AUC in the model including only survey year was 0.58. In the age adjusted model 2, the AUC was 0.60 and it did not increase when gender was included in model 3. The AUC increased by 0.04 units when including education. It did not increase when adding migration status but further increased by 0.01 units when including civil status. The AUC of the intersectional model 7 was 0.65, which is identical to the AUC of model 6 indicating an absence of intersectional interaction.

Table 2 shows the 10 strata with the lowest and the 10 strata with the highest RRs of smoking using the strata of young native men with high educational achievement and cohabiting as reference. The lowest RR= 0.55 was observed in older native women with high educational achievement and cohabiting and the highest RR= 4.45 was observed in young immigrant men with low educational achievement and living alone. When comparing with the reference stratum of native young men with high educational achievement and cohabiting, we observed that low educational achievement, being immigrant and living alone were respectively present in seven, eight and nine of the ten strata with the highest risk of smoking (see the Supplementary information S2 for the complete list of RR-values).

For peer review only

Discussion

Main findings

Our study provides an improved mapping of the distribution of the smoking habit in Sweden compared to unidimensional analyses. Rather than focusing on single socioeconomic and demographical variables, we use an intersectional AIHDA analysis that uncovers the socioeconomic and demographical heterogeneity existing in the country. We also applied the AUC to obtain information on the accuracy of the intersectional grouping for identifying individuals according to their smoking status. We found a moderate AUC= 0.65, which indicates that individual risk of smoking considerably overlaps between the intersectional strata.

We found that the stratum-specific risks were due to the main effects of the different variables used to define the intersectional strata without any interactive component. In fact, the AUC was the same whether the variables were included in the model separately or as a multicategorical variable that allows detection of interaction effects, if they exist.

We found intersectional strata with a rather high RR of smoking. For instance, the prevalence of smoking in young immigrant men with low educational achievement and living alone was 54%. Interestingly, while high educational achievement generally prevents smoking, young immigrant women that lived alone had a relative risk of 2.87 (1.86-4.42) despite their high educational achievement. This indicates that the protective effect of high education may depend on other variables such as migration status and gender. Our finding could hypothetically reflect both smoking culture in the country of birth of the individual or that discrimination on the basis of gender or migration status may contribute to making education a poorer indicator of socioeconomic position in this group.

For peer review only

Relation to previous studies

In spite of the use of different definitions and measurements of smoking habits as well as the use of different indicators of socioeconomic position, many previous publications have shown the existence of socioeconomic, ethnic and demographical differences in smoking [2, 4, 37]. However, as far we known, only a few have considered the intersectional approach [11, 14, 15]. From this perspective our study provides an original contribution. The heterogeneous socioeconomic and demographic distribution of smoking prevalence we found in Sweden is in accordance with recent intersectional research on smoking cessation in the U.S. adult population [15].

Which SEP-indicator that is used influence the hypothesized mediating pathways. In the present study we used educational achievement. High education may influence smoking through both direct effect, such as increased understanding of detrimental health effects of smoking, and indirect effects such as social and material circumstances [38]. The educational gradient, however, might partly be explained by income disparities between people with high or low education [39]. Since income and education capture different aspects of social class, it can be motivated to include both variables in an intersectional matrix. However, we wanted to present a parsimonious model that can be easily applied in public health reports. Educational achievement is the preferred indicator of socioeconomic position in previous public health reports in Sweden [40]. We performed a sensitivity analysis where we included income instead of education and the results were very similar.

In a comparison of the relative importance of low education on smoking prevalence across age- and gender groups in Denmark and Sweden, Eek et al. found that the effect of low education on smoking prevalence and continuation of smoking was strongest among younger women in Sweden, indicating a failure of tobacco prevention interventions to reach this group

For peer review only

[1]. While immigrant men were clearly overrepresented among the strata with highest prevalence of smoking, this was not the case for women. This pattern was also found by Lindström et al in a study from southern Sweden showing lower rates of smoking among men born in Sweden, but higher rates of smoking among women born in Sweden compared to men and women from most other country groups [8]. The distribution of smoking prevalence across age groups we found is similar to the pattern observed by Ali et al in a study from southern Sweden [41].

Strengths and limitations

The cross-sectional and observational character of this study prevents causal conclusions. However, the variables included in our analyses are to a little extent effected by smoking status, so the causal direction can be presumed to go from sociodemographic variables towards smoking rather than the opposite.

Another weakness in our study is that the participation rates were rather low, especially during the last years. An analyses of the non-participants performed by Statistics Sweden shows that people with low income, people born outside Sweden and people living alone were less likely to be responders [42]. Therefore, if the prevalence of smoking is higher in non-participants, our analysis may have underestimated the existing socioeconomic differences. We also performed a sensitivity analysis in which we utilized data that had been weighted by Statistics Sweden in order to reduce skewness resulting from non-participating individuals. The variables used to perform the weighting were age, gender, educational level, country of birth, civil status and urban/rural [43]. This analysis yielded very similar results, which was expected since the intersectional variable included the weighting variables except rural/urban. Finally, our study represents the Swedish circumstances so the AIHDA-approach needs to be replicated in different contexts and for different health outcomes.

For peer review only

Implications and future studies

There is a growing body of literature focusing on how to perform quantitative intersectional research [24, 44], with the emergence of multilevel AIHDA (MAIHDA) as a recent example [25, 26, 30]. However, in spite of providing complementary information [30], the fixed effects AIHDA approach we use in our study is rather accessible and share crucial advantages of the MAIHDA. First, the AIHDA provides an intersectional mapping that is more appropriate than unidimensional analyses to identify specifically vulnerable population groups in which interventions could be effective. Second, by going beyond average probabilistic measurements (i.e., prevalence) and also analysing DA we get a quantification of the heterogeneity around the averages [36]. From the AIHDA we found that the DA of our intersectional model was only moderate which indicates the necessity for universal interventions due to a large unexplained heterogeneity. However, we also identified that the three most vulnerable groups (i.e., strata) included immigrant men with low education younger than 65 years. This finding suggest that special preventive measures should be directed to these groups. Research methods that actively involves members of marginalized groups and has the explicit purpose to result in public health improvements are developing and could be one way forward [45]. In addition to this, availability of smoking cessation aid, political and economic changes to reduce socioeconomic disparities between immigrants and natives are potential strategies.

Conclusions

Compared with studies focused on single variables, the intersectional AIHDA offers a better mapping of the socioeconomic and demographical distribution of smoking in Sweden. However, the moderate DA found in the AIHDA analysis suggested the existence of

For peer review only

substantial unexplained heterogeneity in smoking risk within the different intersectional strata defined by age, gender, education, civil status and migration status. An intersectional AIHDA approach is necessary to understand the existing socioeconomic and demographic complexity influencing smoking behaviour. Future studies should identify preventive measures that are guided by proportionate universalism to find practical ways forwards to reduce intersectional disparities in smoking prevalence.

Footnotes

Funding

This work was supported by grants to Juan Merlo (PI) from the Swedish Research Council (Vetenskapsrådet) for the project *"Multilevel Analyses of Individual Heterogeneity: innovative concepts and methodological approaches in Public Health and Social Epidemiology"* (https://www.swecris.se/betasearch/details/project/201701321VR?lang=en. Project id: 2017-01321.) The study was also supported by a grant for advanced doctoral studies from the Faculty of Medicine at Lund University (Dnr 2020/1125, SAF)

The funders had no role in study design, data collection and analysis, decision to publish, or preparation of the manuscript.

We acknowledge the staff and participants of all the national health surveys that has made this study possible.

Contributors

SAF and JM had the original idea of the study. SAF wrote the initial version of the paper, SAF, JM, RPV and ML participated in the design, analysis, interpretation of data and drafting of the article. All authors approved the final version to be published. JM is the guarantor of the article.

For peer review only

Conflict of interest

There are no conflicts of interest.

Data sharing statement

The original databases from the Public Health Agency of Sweden used in this study are protected by strict rules of confidentiality that cover sensitive individual information including data regarding health and health behaviours. The data can be made available for research after review by a regional Ethics Committee and the Public Health Agency's own safety committee.

Ethics approval

The present investigation was approved by the Swedish Ethical Review Authority (Dnr: 2019-01793) and the data safety committee at the Public Health Agency of Sweden.

License statement

I, the Submitting Author has the right to grant and does grant on behalf of all authors of the Work (as defined in the below author licence), an exclusive licence and/or a non-exclusive licence for contributions from authors who are: i) UK Crown employees; ii) where BMJ has agreed a CC-BY licence shall apply, and/or iii) in accordance with the terms applicable for US Federal Government officers or employees acting as part of their official duties; on a worldwide, perpetual, irrevocable, royalty-free basis to BMJ Publishing Group Ltd ("BMJ") its licensees and where the relevant Journal is co-owned by BMJ to the co-owners of the Journal, to publish the Work in any BMJ products and to exploit all rights, as set out in our licence.

For peer review only

References

[1] Eek F, Ostergren P-O, Diderichsen F*, et al.* Differences in socioeconomic and gender inequalities in tobacco smoking in Denmark and Sweden; a cross sectional comparison of the equity effect of different public health policies. *BMC public health* 2010;**10**:9-9.

[2] Kuntz B, Lampert T. Educational differences in smoking among adolescents in Germany: what is the role of parental and adolescent education levels and intergenerational educational mobility? *International journal of environmental research and public health* 2013;**10**(7):3015-3032.

[3] Härkönen J, Lindberg M, Karlsson L*, et al.* Education is the strongest socio-economic predictor of smoking in pregnancy. *Addiction* 2018;**113**(6):1117-1126.

[4] Hiscock R, Bauld L, Amos A*, et al.* Socioeconomic status and smoking: a review. *Annals of the New York Academy of Sciences* 2012;**1248**(1):107-123.

[5] Graham H. Gender and class as dimensions of smoking behaviour in Britain: insights from a survey of mothers. *Social science & medicine (1982)* 1994;**38**(5):691-698.

[6] Joffer J, Burell G, Bergstrom E*, et al.* Predictors of smoking among Swedish adolescents. *BMC public health* 2014;**14**:1296.

[7] Gilman SE, Martin LT, Abrams DB*, et al.* Educational attainment and cigarette smoking: a causal association? *International journal of epidemiology* 2008;**37**(3):615-624.

[8] Lindstrom M, Sundquist J. Ethnic differences in daily smoking in Malmo, Sweden. Varying influence of psychosocial and economic factors. *European journal of public health* 2002;**12**(4):287-294.

[9] Lindstrom M, Giordano GN. Changes in Social Capital and Cigarette Smoking Behavior Over Time: A Population-Based Panel Study of Temporal Relationships. *Nicotine & tobacco research : official journal of the Society for Research on Nicotine and Tobacco* 2016;**18**(11):2106-2114.

[10] Ramstrom L, Borland R, Wikmans T. Patterns of Smoking and Snus Use in Sweden: Implications for Public Health. *Int J Environ Res Public Health* 2016;**13**(11).

[11] Amroussia N, Pearson JL, Gustafsson PE. What drives us apart? Decomposing intersectional inequalities in cigarette smoking by education and sexual orientation among U.S. adults. *International journal for equity in health* 2019;**18**(1):109.

[12] Mena E, Bolte G, Group AGS. Intersectionality-based quantitative health research and sex/gender sensitivity: a scoping review. *International journal for equity in health* 2019;**18**(1):199-199.

[13] Bilal U, Beltrán P, Fernández E*, et al.* Gender equality and smoking: a theory-driven approach to smoking gender differences in Spain. *Tobacco control* 2016;**25**(3):295-300.

[14] Aguirre CG, Bello MS, Andrabi N*, et al.* Gender, Ethnicity, and Their Intersectionality in the Prediction of Smoking Outcome Expectancies in Regular Cigarette Smokers. *Behavior modification* 2016;**40**(1-2):281-302.

[15] Potter LN, Lam CY, Cinciripini PM*, et al.* Intersectionality and Smoking Cessation: Exploring Various Approaches for Understanding Health Inequities. *Nicotine & Tobacco Research* 2020.

[16] Vilhelmsson A, Ostergren PO. Reducing health inequalities with interventions targeting behavioral factors among individuals with low levels of education - A rapid review. *PloS one* 2018;**13**(4):e0195774.

For peer review only

[17] Wendel-Vos GC, Dutman AE, Verschuren WM, *et al.* Lifestyle factors of a five-year community-intervention program: the Hartslag Limburg intervention. *American journal of preventive medicine* 2009;**37**(1):50-56.
[18] Marmot M, Bell R. Fair society, healthy lives. *Public health* 2012;**126 Suppl 1**:S4-s10.
[19] Carey G, Crammond B, De Leeuw E. Towards health equity: a framework for the application of proportionate universalism. *International journal for equity in health* 2015;**14**:81.
[20] Wemrell M, Bennet L, Merlo J. Understanding the complexity of socioeconomic disparities in type 2 diabetes risk: a study of 4.3 million people in Sweden. *MJ Open Diabetes Research and Care* 2019;**9;7:e000749.**
[21] Merlo J, Wagner P, Leckie G. A simple multilevel approach for analysing geographical inequalities in public health reports: The case of municipality differences in obesity. *Health & place* 2019;**58**:102145.
[22] K C. Demarginalizing the intersection of race and sex: a Black feminist critique of antidiscriminiation doctrine, feminist theory and antiracist politics. *University of Chicago Legal Forum* 1989(139).
[23] Bauer GR. Incorporating intersectionality theory into population health research methodology: challenges and the potential to advance health equity. *Soc Sci Med* 2014;**110**:10-17.
[24] Bowleg L. When Black plus Lesbian plus Woman not equal Black Lesbian Woman: The methodological challenges of qualitative and quantitative intersectionality research. *Sex Roles* 2008;**59**(5-6):312-325.
[25] Merlo J. Multilevel analysis of individual heterogeneity and discriminatory accuracy (MAIHDA) within an intersectional framework. *Social science & medicine (1982)* 2018;**203**:74-80.
[26] Wemrell M, Mulinari S, Merlo J. An intersectional approach to multilevel analysis of individual heterogeneity (MAIH) and discriminatory accuracy. *Social science & medicine (1982)* 2017;**178**:217-219.
[27] Axelsson Fisk S, Mulinari S, Wemrell M, *et al.* Chronic Obstructive Pulmonary Disease in Sweden: An intersectional multilevel analysis of individual heterogeneity and discriminatory accuracy. *SSM Popul Health* 2018;**4**:334-346.
[28] Persmark A, Wemrell M, Zettermark S, *et al.* Precision public health: Mapping socioeconomic disparities in opioid dispensations at Swedish pharmacies by Multilevel Analysis of Individual Heterogeneity and Discriminatory Accuracy (MAIHDA). *PLoS One* 2019;**14**(8):e0220322.
[29] Evans CR, Erickson N. Intersectionality and depression in adolescence and early adulthood: A MAIHDA analysis of the national longitudinal study of adolescent to adult health, 1995-2008. *Soc Sci Med* 2019;**220**:1-11.
[30] Evans CR, Leckie G, Merlo J. Multilevel versus single-level regression for the analysis of multilevel information: The case of quantitative intersectional analysis. *Social science & medicine (1982)* 2019:112499.
[31] Evans CR, Williams DR, Onnela JP, *et al.* A multilevel approach to modeling health inequalities at the intersection of multiple social identities. *Soc Sci Med* 2017.
[32] Davies HT, Crombie IK, Tavakoli M. When can odds ratios mislead? *BMJ (Clinical research ed)* 1998;**316**(7136):989-991.
[33] Barros AJ, Hirakata VN. Alternatives for logistic regression in cross-sectional studies: an empirical comparison of models that directly estimate the prevalence ratio. *BMC Med Res Methodol* 2003;**3**:21.

For peer review only

[34] Pepe MS, Janes H, Longton G, *et al.* Limitations of the odds ratio in gauging the performance of a diagnostic, prognostic, or screening marker. *American journal of epidemiology* 2004;**159**(9):882-890.
[35] Hosmer DW, Lemeshow S. Applied logistic regression. 2nd ed. New York: Wiley 2000.
[36] Merlo J, Wagner P, Leckie G. A simple multilevel approach for analysing geographical inequalities in public health reports: The case of municipality differences in obesity. *Health Place* 2019;**58**:102145.
[37] Landberg J, Ramstedt M, Sundin E. Socioekonomiska skillnader i beroende och utsatthet för andras användning av alkohol, narkotika och tobak. Stockholm: Centralförbundet för alkohol- och narkotikaupplysning 2018.
[38] Östergren O. Understanding the educational gradient in Mortality: Department of Sociology, Stockholm University 2017.
[39] Cutler DM, Lleras-Muney A. Understanding differences in health behaviors by education. *Journal of health economics* 2010;**29**(1):1-28.
[40] Folkhälsomyndigheten SkoR. Regional comparisons public health 2019. www.folkhalsomyndigheten.se/publicerat-material 2018.
[41] Ali SM, Chaix B, Merlo J, *et al.* Gender differences in daily smoking prevalence in different age strata: A population-based study in southern Sweden. *Scandinavian journal of public health* 2009;**37**(2):146-152.
[42] Franzén M. Extra bortfallsanalys. Statistics Sweden 2018.
[43] Franzén M. Kalibreringsrapport Nationellt Urval. Stockholm: Statistics Sweden 2015.
[44] McCall L. The complexity of intersectionality. *Signs: Journal of women in culture and society* 2005;**30**(3):1771-1800.
[45] Andrews JO, Newman SD, Heath J, *et al.* Community-based participatory research and smoking cessation interventions: a review of the evidence. *Nursing Clinics* 2012;**47**(1):81-96.

For peer review only

Tables

Table 1. Distribution (prevalence) of smokers across categories of age, gender, education, migration and civil status in the 110,044 participants in the Swedish National Health Surveys (2004 – 2018). Values are number (and percentage) of individuals.

	Non smokers	Smokers
30-44	22,799 (80.23%)	5,618 (19.77%)
45-64	38,024 (79.41%)	9,862 (20.59%)
65-84	29,575 (87.65%)	4,166 (12.35%)
Female	48,782 (82.08%)	10.653 (17.92%)
Male	41,616 (82.23%)	8,993 (17.77%)
Low	38,791 (78.32%)	10,738 (21.68%)
Middle	27,716 (83.02%)	5,670 (16.98)
High	23,891 (88.06%)	3,238 (11.94%)
Immigrant	10,410 (76.07%)	3,274 (23.93%)
Native	79,988 (83.01%)	16,372 (16.99%)
Cohabiting	75,625 (83.48%)	14,964 (16.52%)
Living Alone	14,773 (75.93%)	4,682 (24.07%)
2004	6,803 (75.03%)	2,264 (24.97%)
2005	3,339 (75.90%)	1,060 (24.10%)
2006	3,450 (77.62%)	995 (22.38%)
2007	3,272 (77.81%)	933 (22.19%)
2008	6,525 (79.07%)	1,727 (20.93%)
2009	6,123 (79.22%)	1,606 (20.78%)
2010	6,718 (80.59%)	1,618 (19.41%)
2011	6,760 (82.56%)	1,428 (17.44%)
2012	6,893 (82.68%)	1,444 (17.32%)
2013	6,770 (83.10%)	1,377 (16.90%)
2014	6,845 (83.74%)	1,329 (16.26%)
2015	6,978 (84.21%)	1,308 (15.79%)
2016	7,086 (88.13%)	954 (11.87%)
2018	12,836 (88.90%)	1,603 (11.10%)

For peer review only

Table 2. Results from the intersectional model 7 indicating the 10 highest and 10 lowest Relative risk (RR) with 95% confidence intervals (CI) of smoking across intersectional strata in the Swedish population using the stratum of young, native, men with high education that were cohabiting as reference in the comparisons.

Age	Gender	Educational achievement	Migration status	Civil status	RR (95% CI)
65-84	Female	High	Native	Cohabiting	0.55 (0.45-0.69)
65-84	Male	High	Native	Cohabiting	0.58 (0.48-0.71)
65-84	Female	High	Immigrant	Cohabiting	0.61 (0.33-1.11)
65-84	Female	Middle	Native	Cohabiting	0.80 (0.66-0.96)
65-84	Female	High	Native	Living alone	0.83 (0.64-1.06)
65-84	Male	Middle	Native	Cohabiting	0.85 (0.73-0.99)
30-44	Female	High	Native	Cohabiting	0.86 (0.74-0.98)
65-84	Male	High	Immigrant	Living alone	0.91 (0.38-2.21)
45-64	Male	High	Native	Cohabiting	0.92 (0.8-1.07)
65-84	Male	Low	Native	Cohabiting	0.96 (0.84-1.11)
30-44	**Male**	**High**	**Native**	**Cohabiting**	**Reference**
30-44	Female	High	Immigrant	Living alone	2.87 (1.86-4.42)
30-44	Female	Low	Native	Living alone	2.95 (2.29-3.78)
45-64	Female	Low	Native	Living alone	2.99 (2.61-3.41)
45-64	Male	Middle	Immigrant	Living alone	3.10 (2.26-4.26)
45-64	Female	Low	Immigrant	Living alone	3.22 (2.56-4.06)
30-44	Male	Middle	Immigrant	Living alone	3.33 (2.35-4.71)
30-44	Female	Low	Immigrant	Living alone	3.41 (1.96-5.94)
45-64	Male	Low	Immigrant	Living alone	3.61 (2.90-4.50)
30-44	Male	Low	Immigrant	Cohabiting	3.66 (3.07-4.35)
30-44	Male	Low	Immigrant	Living alone	4.45 (3.29-6.03)
AUC					0.65
ΔAUC compared with model 6					0

For peer review only

Table 3. Relative risks (RR) and 95%-confidence intervals (CI), of smoking among people aged 30-84 included in the National Health Surveys between 2004 and 2018 in relation to survey year, age, gender, education, migration- and civil status. Model 6 includes the same variables as model 5 but as a multicategorical variable, The RRs for model 7 are presented in the table 3. AUC-values with 95%CI representing the discriminatory accuracy and ΔAUC-values of the models are also presented.

		Model 1	Model 2	Model 3	Model 4	Model 5	Model 6
Year							
	2004	2.25 (2.11-2.40)	2.07 (1.95-2.21)	2.08 (1.95-2.21)	1.85 (1.74-1.98)	1.88 (1.76-2.00)	1.84 (1.73-1.97)
	2005	2.17 (2.01-2.35)	2.01 (1.86-2.17)	2.01 (1.86.2.17)	1.83 (1.69.1.98)	1.85 (1.71-2.00)	1.83 (1.69-1.98)
	2006	2.02 (1.86-2.18)	1.87 (1.72-2.02)	1.87 (1.72-2.02)	1.70 (1.57-1.84)	1.71 (1.58-1.85)	1.68 (1.55-1.82)
	2007	2.00 (1.84 -2.17)	1.86 (1.71.2.01)	1.86 (1.71-2.01)	1.71 (1.57-1.85)	1.65 (1.54-1.77)	1.70 (1.56.1.84)
	2008	1.89 (1.76-2.02)	1.76 (1.64-1.88)	1.76 (1.64-1.88)	1.64 (1.53-1.75)	1.66 (1.55-1.78)	1.63 (1,52-1.74)
	2009	1.87 (1.75-2.01)	1.75 (1.64-1.89)	1.76 (1.64-1-88)	1.65 (1.54-1.77)	1.62 (1.51-1.73)	1.63 (1.52-1.75)
	2010	1.75 (1.63-1.87)	1.70 (1.58-1.82)	1.70 (1.58-1.82)	1.61 (1.50-1.72)	1.46 (1.36-1-57)	1.59 (1.48-1.70)
	2011	1.57 (1.46-1.69)	1.53 (1.43-1.64)	1.53 (1.43-1-64)	1.45 (1.35-1.56)	1.47 (1.37-1.58)	1.43 (1.33-1.54)
	2012	1.56 (1.45-1.68)	1.53 (1.42-1.64)	1.53 (1.42-1.64)	1.47 (1.37-1.57)	1.45 (1.35-1.56)	1.44 (1.34-1.55)
	2013	1.52 (1-42-1.64)	1.49 (1.39-1.60)	1.49 (1.39-1.60)	1.45 (1.35-1.55)	1.45 (1.35-1.56)	1.42 (1.32-1.52)
	2014	1.47 (1.36-1.75)	1.45 (1.35-1.56)	1.45 (1.35-1.56)	1.41 (1.31-1.52)	1.42 (1.32-1.53)	1.39 (1.30-1.50)
	2015	1.42 (1.32-1.53)	1.40 (1.30-1.51)	1.40 (1.30-1.51)	1.37 (1.27-1.47)	1.38 (1.28-1.48)	1.35 (1.26-1.46)
	2016	1.07 (0.99-1.16)	1.06 (0.97-1.14)	1.06 (0.97-1.14)	1.04 (0.96-1.13)	1.05 (0.97-1.13)	1. 03 (0.95-1.11)
	2018	Reference	Reference	Reference	Reference	Reference	Reference
Age							
	30-44		Reference	Reference	Reference	Reference	Reference
	45-64		1.06 (1.0.3-1.10)	1.06 (1.03-1.10)	0.94 (0.91-0.97)	0.95 (0.91-0.98)	0.93 (0.90-0.96)
	65-84		0.68 (0.65-0.71)	0.68 (0.65-0.71)	0.56 (0.54-0.58)	0.57 (0.55-0.59)	0.53 (0.51-0.56)
Gender							
	Male			Reference	Reference	Reference	Reference
	Female			1.00 (0.97-1.02)	1.02 (0.99-1.05)	1.02 (0.99-1.05)	1.01 (0.98-1.04)
Education							
	Low				1.96 (1.88-2.04)	1.96 (1.88-2.04)	1.93 (1.86-2.01)
	Middle				1.42 (1.36-1.48)	1.43 (1.37-1.49)	1.42 (1.36-1.49)
	High				Reference	Reference	Reference

For peer review only

Born in Sweden							
	Native					Reference	Reference
	Immigrant					1.39 (1.34-1.45)	1.39 (1.24-1.44)
Living alone							
	Living alone						1.53 (1.48-1.58)
	cohabiting						Reference
AUC		0.58 (0.58-0.59)	0.60 (0.60-0.61)	0.60 (0.60-0.61)	0.64 (0.63-0.64)	0.64 (0.64-0.65)	0.65 (0.65-0.66)
ΔAUC		-	0.02	0.00	0.04	0.00	0.01

For peer review only

Legend to the figures

Figure 1. Flow chart showing the selection of the study population.

Figure 2. Absolute risk (i.e., prevalence) and 99% confidence intervals of smoking in different intersectional strata according the National Health Survey in Sweden between 2004 and 2018.

For peer review only

For peer review only

Figure 1. Flow chart showing the selection of the study population.

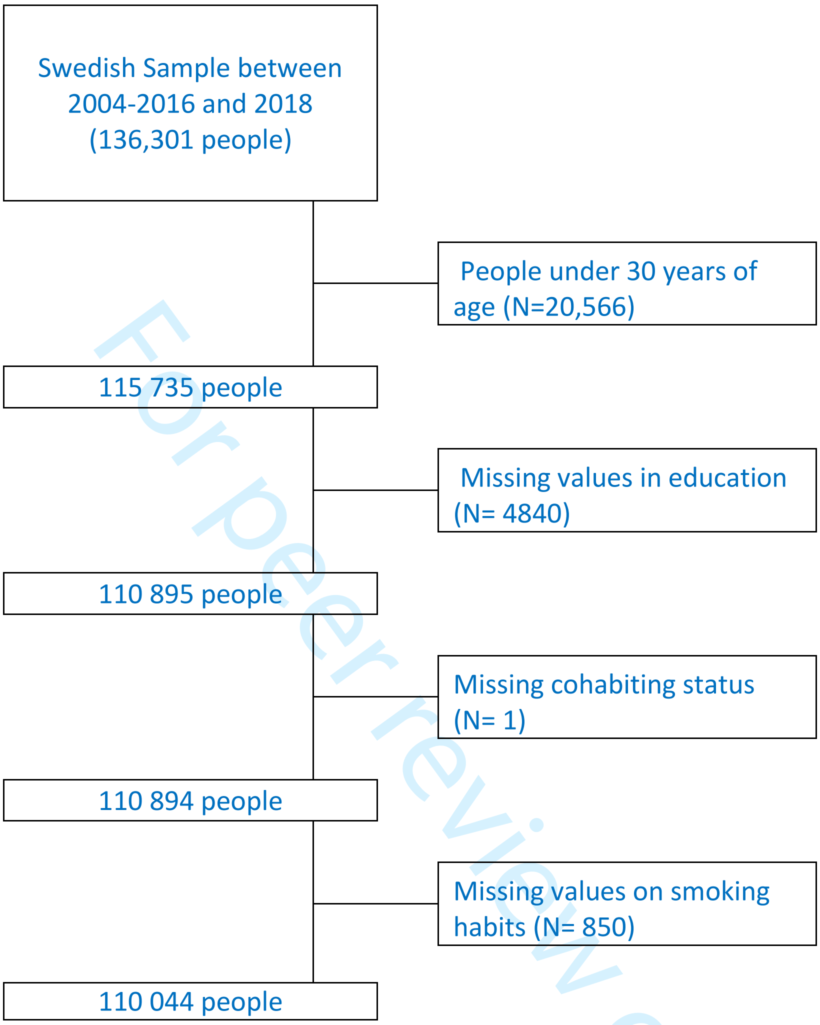

For peer review only

Figure 2. Absolute risk (i.e., prevalence) and 99% confidence intervals of smoking in different intersectional strata according the National Health Survey in Sweden between 2004 and 2018.

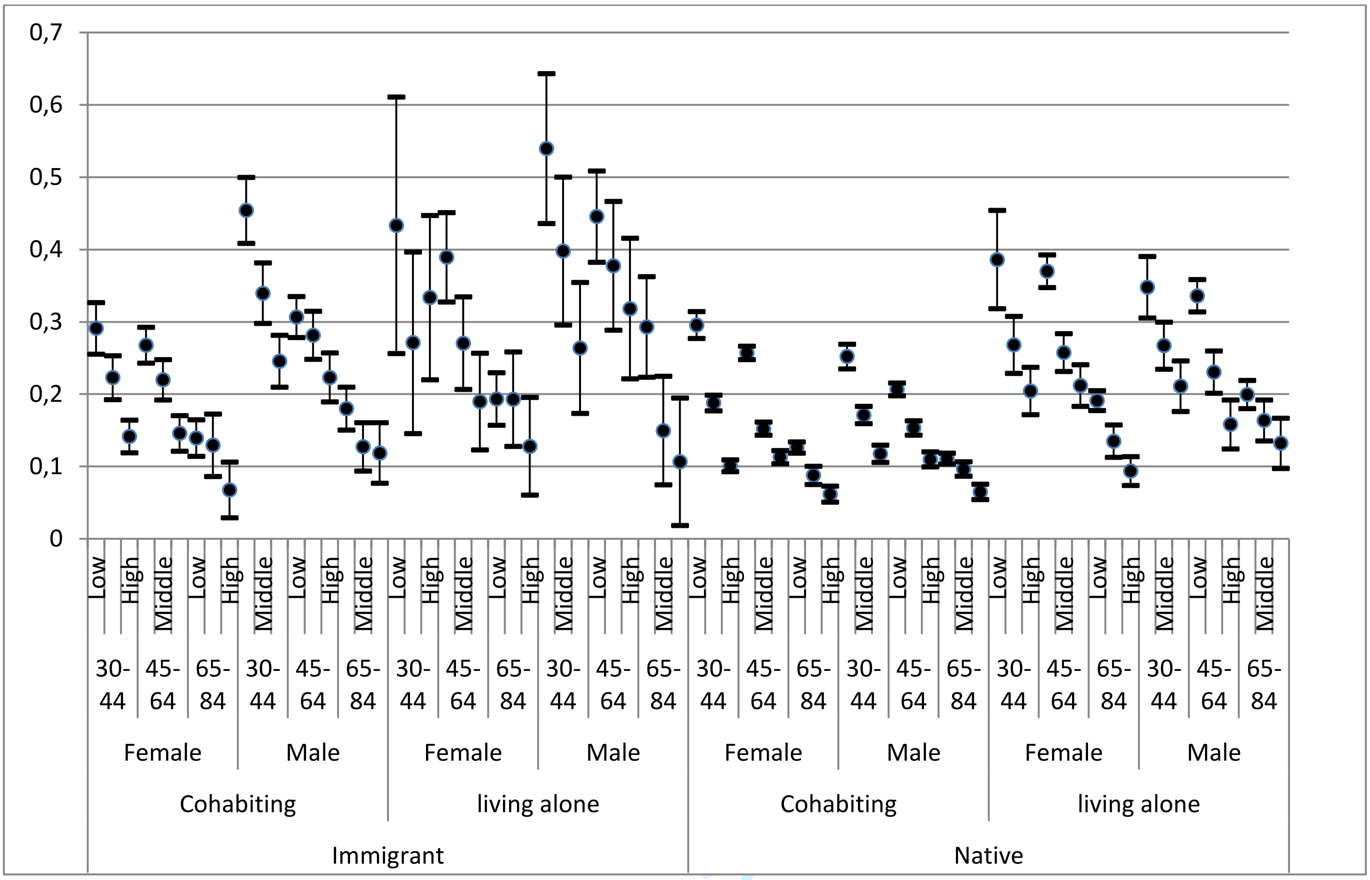

Supplementary material 1

S1

Trends in smoking prevalence in the National Health Surveys for Sweden. Categories of age, gender, educational achievement, migration status and civil status are shown with different lines in the respective graphs.

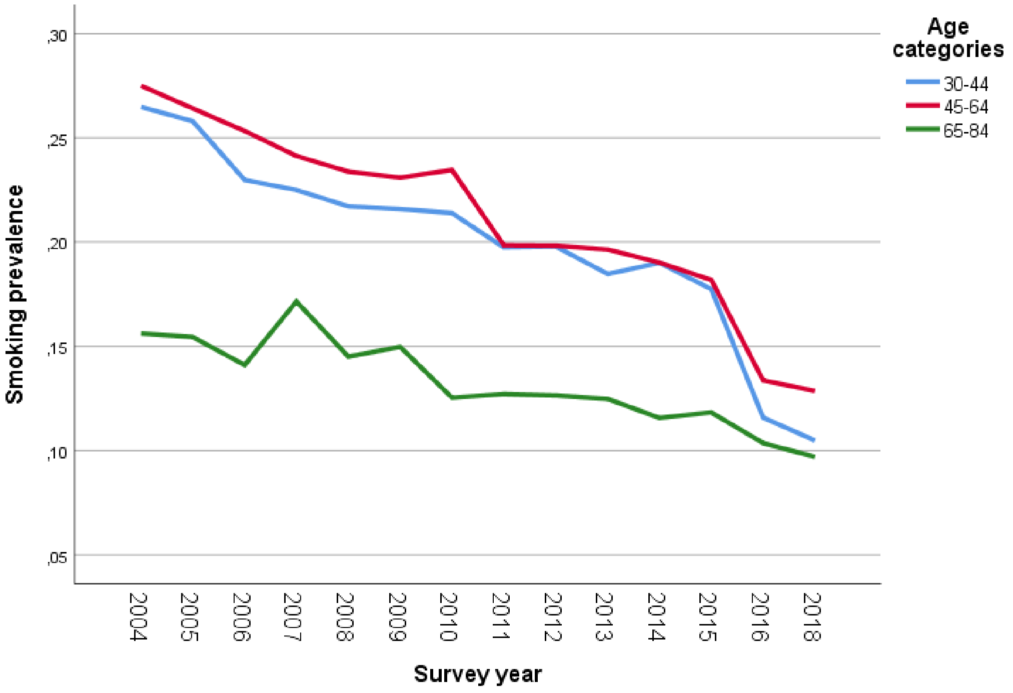

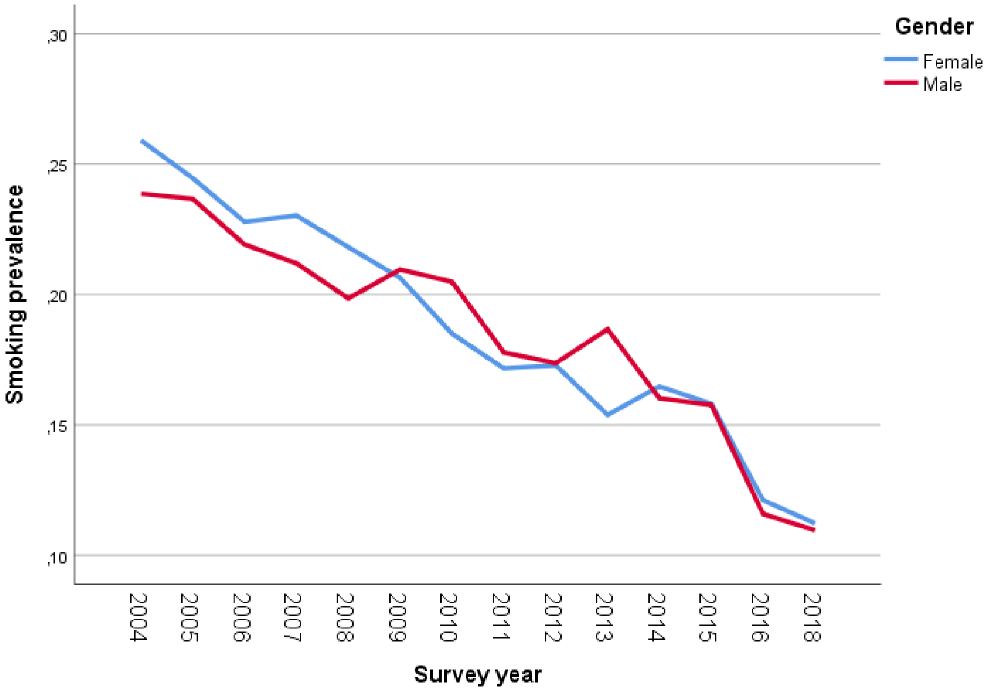

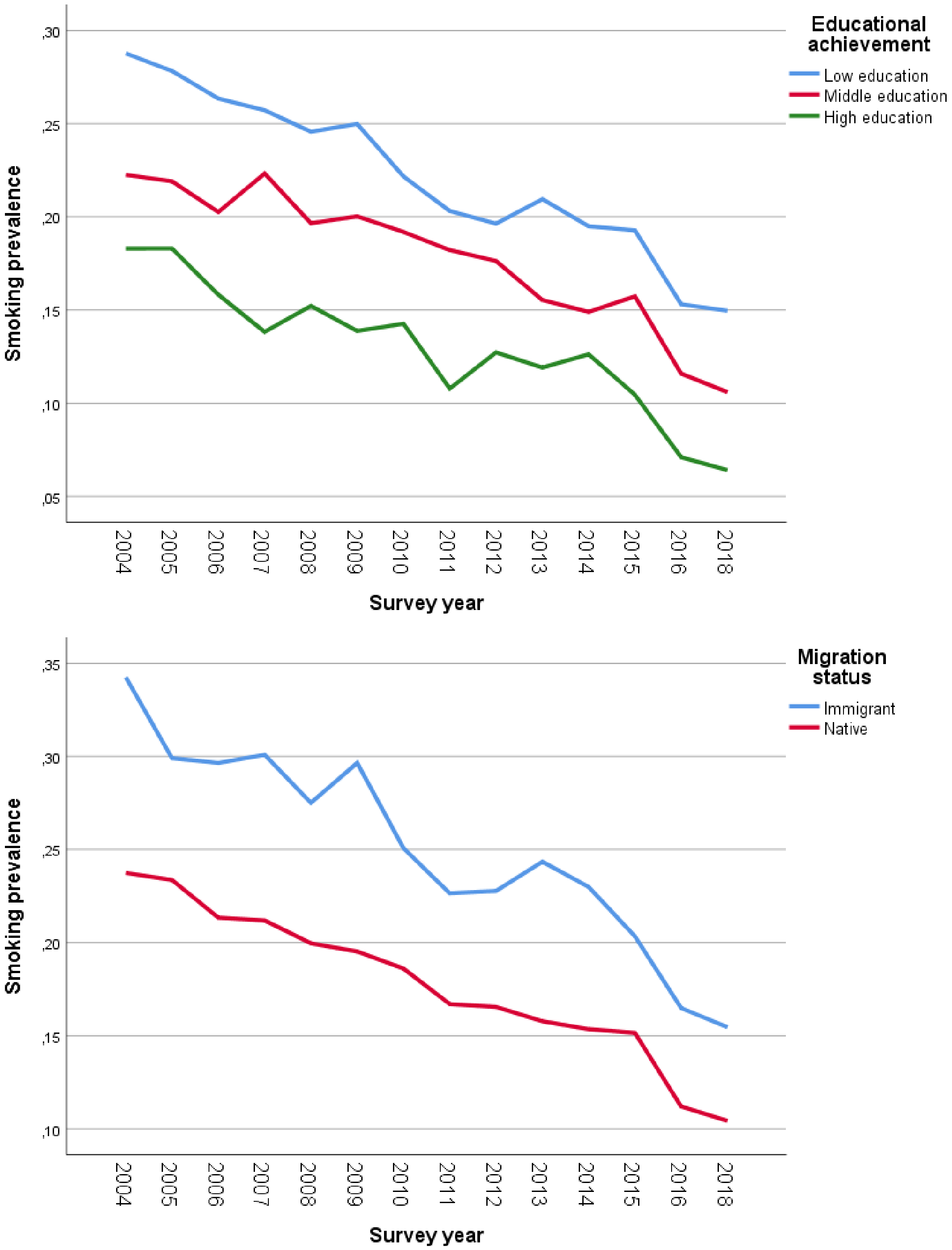
Educational achievement
Low education
Middle education
High education
Smoking prevalence
,30
,25
,20
,15
,10
,05
2004
2005
2006
2007
2008
2009
2010
2011
2012
2013
2014
2015
2016
2018
Survey year
Migration status
Immigrant
Native
Smoking prevalence
,35
,30
,25
,20
,15
,10
2004
2005
2006
2007
2008
2009
2010
2011
2012
2013
2014
2015
2016
2018
Survey year

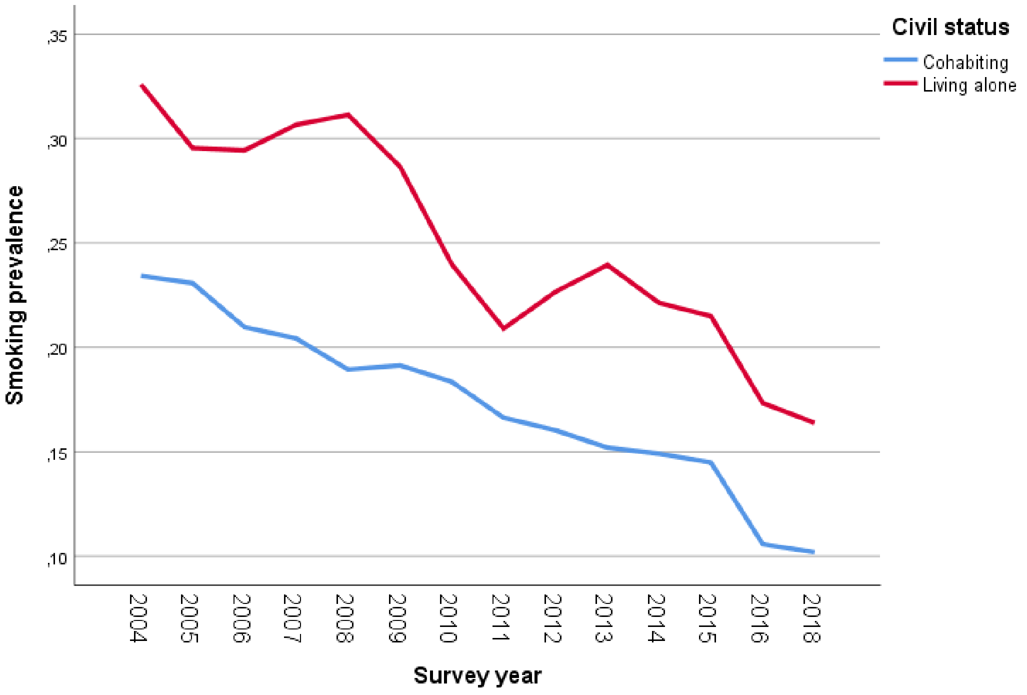

For peer review only

For peer review only

Supplementary material 2

S2

Table 2, full version. Results from the intersectional model 7 indicating the 10 highest and 10 lowest Relative risk (RR) with 95% confidence intervals (CI) of smoking across intersectional strata in the Swedish population using the stratum of young, native, men with high education that were cohabiting as reference in the comparisons. The area under the receiver operator characteristic curve (AUC) values with 95%CI and the increment in the AUC (ΔAUC) values in successive models are also presented.

Age	Gender	Educational achievement	Migration status	Civil status	RR (95% CI)
30-44	Female	Low	Immigrant	Cohabiting	2.35 (1.96-2.82)
30-44	Female	Low	Immigrant	Living alone	3.41 (1.96-5.94)
30-44	Female	Low	Native	Cohabiting	2.24 (1.96-2.56)
30-44	Female	Low	Native	Living alone	2.95 (2.29-3.78)
30-44	Female	Middle	Immigrant	Cohabiting	1.83 (1.51-2.21)
30-44	Female	Middle	Immigrant	Living alone	2.33 (1.34-4.05)
30-44	Female	Middle	Native	Cohabiting	1.53 (1.35-1.73)
30-44	Female	Middle	Native	Living alone	2.2 (1.8-2.7)
30-44	Female	High	Immigrant	Cohabiting	1.22 (0.99-1.49)
30-44	Female	High	Immigrant	Living alone	2.87 (1.86-4.42)
30-44	Female	High	Native	Cohabiting	0.86 (0.74-0.98)
30-44	Female	High	Native	Living alone	1.72 (1.39-2.12)
30-44	Male	Low	Immigrant	Cohabiting	3.66 (3.07-4.35)
30-44	Male	Low	Immigrant	Living alone	4.45 (3.29-6.03)
30-44	Male	Low	Native	Cohabiting	1.92 (1.68-2.2)
30-44	Male	Low	Native	Living alone	2.67 (2.21-3.21)
30-44	Male	Middle	Immigrant	Cohabiting	2.84 (2.36-3.43)
30-44	Male	Middle	Immigrant	Living alone	3.33 (2.35-4.71)
30-44	Male	Middle	Native	Cohabiting	1.43 (1.25-1.63)
30-44	Male	Middle	Native	Living alone	2.21 (1.85-2.64)
30-44	Male	High	Immigrant	Cohabiting	2.13 (1.74-2.6)
30-44	Male	High	Immigrant	Living alone	2.32 (1.53-3.5)

For peer review only

30-44	Male	High	Native	Cohabiting	Reference
30-44	Male	High	Native	Living alone	1.75 (1.41-2.18)
45-64	Female	Low	Immigrant	Cohabiting	2.19 (1.88-2.55)
45-64	Female	Low	Immigrant	Living alone	3.22 (2.56-4.06)
45-64	Female	Low	Native	Cohabiting	2.08 (1.85-2.34)
45-64	Female	Low	Native	Living alone	2.99 (2.61-3.41)
45-64	Female	Middle	Immigrant	Cohabiting	1.87 (1.56-2.23)
45-64	Female	Middle	Immigrant	Living alone	2.23 (1.66-3.01)
45-64	Female	Middle	Native	Cohabiting	1.29 (1.14-1.46)
45-64	Female	Middle	Native	Living alone	2.16 (1.84-2.53)
45-64	Female	High	Immigrant	Cohabiting	1.27 (1.03-1.57)
45-64	Female	High	Immigrant	Living alone	1.63 (1.08-2.45)
45-64	Female	High	Native	Cohabiting	0.97 (0.84-1.11)
45-64	Female	High	Native	Living alone	1.76 (1.46-2.13)
45-64	Male	Low	Immigrant	Cohabiting	2.55 (2.18-2.98)
45-64	Male	Low	Immigrant	Living alone	3.61 (2.9-4.5)
45-64	Male	Low	Native	Cohabiting	1.71 (1.52-1.93)
45-64	Male	Low	Native	Living alone	2.77 (2.42-3.17)
45-64	Male	Middle	Immigrant	Cohabiting	2.39 (2.01-2.86)
45-64	Male	Middle	Immigrant	Living alone	3.1 (2.26-4.26)
45-64	Male	Middle	Native	Cohabiting	1.28 (1.12-1.45)
45-64	Male	Middle	Native	Living alone	1.92 (1.61-2.31)
45-64	Male	High	Immigrant	Cohabiting	1.91 (1.56-2.35)
45-64	Male	High	Immigrant	Living alone	2.7 (1.84-3.98)
45-64	Male	High	Native	Cohabiting	0.92 (0.8-1.07)
45-64	Male	High	Native	Living alone	1.35 (1.04-1.75)
65-84	Female	Low	Immigrant	Cohabiting	1.23 (0.98-1.54)
65-84	Female	Low	Immigrant	Living alone	1.62 (1.28-2.05)
65-84	Female	Low	Native	Cohabiting	1.1 (0.97-1.25)

For peer review only

65-84	Female	Low	Native	Living alone	1.62 (1.42-1.86)
65-84	Female	Middle	Immigrant	Cohabiting	1.18 (0.81-1.71)
65-84	Female	Middle	Immigrant	Living alone	1.66 (1.12-2.46)
65-84	Female	Middle	Native	Cohabiting	0.8 (0.66-0.96)
65-84	Female	Middle	Native	Living alone	1.2 (0.97-1.48)
65-84	Female	High	Immigrant	Cohabiting	0.61 (0.33-1.11)
65-84	Female	High	Immigrant	Living alone	1.16 (0.65-2.07)
65-84	Female	High	Native	Cohabiting	0.55 (0.45-0.69)
65-84	Female	High	Native	Living alone	0.83 (0.64-1.06)
65-84	Male	Low	Immigrant	Cohabiting	1.57 (1.27-1.95)
65-84	Male	Low	Immigrant	Living alone	2.49 (1.84-3.37)
65-84	Male	Low	Native	Cohabiting	0.96 (0.84-1.1)
65-84	Male	Low	Native	Living alone	1.71 (1.47-2)
65-84	Male	Middle	Immigrant	Cohabiting	1.12 (0.82-1.51)
65-84	Male	Middle	Immigrant	Living alone	1.29 (0.74-2.24)
65-84	Male	Middle	Native	Cohabiting	0.85 (0.73-0.99)
65-84	Male	Middle	Native	Living alone	1.47 (1.18-1.83)
65-84	Male	High	Immigrant	Cohabiting	1.06 (0.72-1.57)
65-84	Male	High	Immigrant	Living alone	0.91 (0.38-2.21)
65-84	Male	High	Native	Cohabiting	0.58 (0.48-0.71)
65-84	Male	High	Native	Living alone	1.19 (0.88-1.6)

For peer review only

For peer review only

STROBE Statement for manuscript entitled "Understanding the Complexity of Socioeconomic Disparities in smoking prevalence: Combining Intersectionality theory with analysis of individual heterogeneity and discriminatory accuracy (AIHDA)"

	Item No	Recommendation	
**Title and abstract**	1	(*a*) Indicate the study's design with a commonly used term in the title or the abstract	✓
		(*b*) Provide in the abstract an informative and balanced summary of what was done and what was found	✓
**Introduction**			
Background/rationale	2	Explain the scientific background and rationale for the investigation being reported	✓
Objectives	3	State specific objectives, including any prespecified hypotheses	✓
**Methods**			
Study design	4	Present key elements of study design early in the paper	✓
Setting	5	Describe the setting, locations, and relevant dates, including periods of recruitment, exposure, follow-up, and data collection	✓
Participants	6	(*a*) Give the eligibility criteria, and the sources and methods of selection of participants	✓
Variables	7	Clearly define all outcomes, exposures, predictors, potential confounders, and effect modifiers. Give diagnostic criteria, if applicable	✓
Data sources/ measurement	8*	For each variable of interest, give sources of data and details of methods of assessment (measurement). Describe comparability of assessment methods if there is more than one group	✓
Bias	9	Describe any efforts to address potential sources of bias	✓
Study size	10	Explain how the study size was arrived at	✓
Quantitative variables	11	Explain how quantitative variables were handled in the analyses. If applicable, describe which groupings were chosen and why	✓
Statistical methods	12	(*a*) Describe all statistical methods, including those used to control for confounding	✓
		(*b*) Describe any methods used to examine subgroups and interactions	✓
		(*c*) Explain how missing data were addressed	✓
		(*d*) If applicable, describe analytical methods taking account of sampling strategy	✓
		(*e*) Describe any sensitivity analyses	✓
**Results**			
Participants	13*	(a) Report numbers of individuals at each stage of study—eg numbers potentially eligible, examined for eligibility, confirmed eligible, included in the study, completing follow-up, and analysed	✓
		(b) Give reasons for non-participation at each stage	✓
		(c) Consider use of a flow diagram	✓
Descriptive data	14*	(a) Give characteristics of study participants (eg demographic, clinical, social) and information on exposures and potential confounders	✓
		(b) Indicate number of participants with missing data for each variable of interest	✓
Outcome data	15*	Report numbers of outcome events or summary measures	✓

For peer review only - http://bmjopen.bmj.com/site/about/guidelines.xhtml

Main results	16	(*a*) Give unadjusted estimates and, if applicable, confounder-adjusted estimates and their precision (eg, 95% confidence interval). Make clear which confounders were adjusted for and why they were included	✓
		(*b*) Report category boundaries when continuous variables were categorized	✓
		(*c*) If relevant, consider translating estimates of relative risk into absolute risk for a meaningful time period	✓
Other analyses	17	Report other analyses done—eg analyses of subgroups and interactions, and sensitivity analyses	✓
**Discussion**			
Key results	18	Summarise key results with reference to study objectives	✓
Limitations	19	Discuss limitations of the study, taking into account sources of potential bias or imprecision. Discuss both direction and magnitude of any potential bias	✓
Interpretation	20	Give a cautious overall interpretation of results considering objectives, limitations, multiplicity of analyses, results from similar studies, and other relevant evidence	✓
Generalisability	21	Discuss the generalisability (external validity) of the study results	✓
**Other information**			
Funding	22	Give the source of funding and the role of the funders for the present study and, if applicable, for the original study on which the present article is based	✓

*Give information separately for exposed and unexposed groups.

Note: An Explanation and Elaboration article discusses each checklist item and gives methodological background and published examples of transparent reporting. The STROBE checklist is best used in conjunction with this article (freely available on the Web sites of PLoS Medicine at http://www.plosmedicine.org/, Annals of Internal Medicine at http://www.annals.org/, and Epidemiology at http://www.epidem.com/). Information on the STROBE Initiative is available at www.strobe-statement.org.

For peer review only

BMJ Open

Understanding the complexity of socioeconomic disparities in smoking prevalence in Sweden: a cross-sectional study applying intersectionality theory

Journal:	*BMJ Open*
Manuscript ID	bmjopen-2020-042323.R1
Article Type:	Original research
Date Submitted by the Author:	19-Dec-2020
Complete List of Authors:	Axelsson Fisk, Sten; Lund University, Unit for Social Epidemiology, Department of Clinical Sciences<br>Lindstrom, Martin; Lunds University Faculty of Medicine, Unit for Social Medicine and Health Policy, Department of Clinical Sciences; Region Skåne, Centre for Primary Health Care Research<br>Perez-Vicente, Raquel; Lunds University Faculty of Medicine, Unit for Social Epidemiology, Department of Clinical Sciences<br>Merlo, Juan; Lunds University Faculty of Medicine, Unit for Social Epidemiology, Department of Clinical Sciences; Region Skåne, Malmö, Centre for Primary Health Care Research
**Primary Subject Heading**:	Epidemiology
Secondary Subject Heading:	Smoking and tobacco, Public health, Health policy
Keywords:	SOCIAL MEDICINE, EPIDEMIOLOGY, Health policy < HEALTH SERVICES ADMINISTRATION & MANAGEMENT, PUBLIC HEALTH, STATISTICS & RESEARCH METHODS, Substance misuse < PSYCHIATRY

SCHOLARONE™
Manuscripts

I, the Submitting Author has the right to grant and does grant on behalf of all authors of the Work (as defined in the below author licence), an exclusive licence and/or a non-exclusive licence for contributions from authors who are: i) UK Crown employees; ii) where BMJ has agreed a CC-BY licence shall apply, and/or iii) in accordance with the terms applicable for US Federal Government officers or employees acting as part of their official duties; on a worldwide, perpetual, irrevocable, royalty-free basis to BMJ Publishing Group Ltd ("BMJ") its licensees and where the relevant Journal is co-owned by BMJ to the co-owners of the Journal, to publish the Work in this journal and any other BMJ products and to exploit all rights, as set out in our licence.

The Submitting Author accepts and understands that any supply made under these terms is made by BMJ to the Submitting Author unless you are acting as an employee on behalf of your employer or a postgraduate student of an affiliated institution which is paying any applicable article publishing charge ("APC") for Open Access articles. Where the Submitting Author wishes to make the Work available on an Open Access basis (and intends to pay the relevant APC), the terms of reuse of such Open Access shall be governed by a Creative Commons licence – details of these licences and which Creative Commons licence will apply to this Work are set out in our licence referred to above.

Other than as permitted in any relevant BMJ Author's Self Archiving Policies, I confirm this Work has not been accepted for publication elsewhere, is not being considered for publication elsewhere and does not duplicate material already published. I confirm all authors consent to publication of this Work and authorise the granting of this licence.

For peer review only

Understanding the complexity of socioeconomic disparities in smoking prevalence in Sweden: a cross-sectional study applying intersectionality theory

Running Title: Complex Disparities in smoking prevalence

Journal BMJ-Open

Sten Axelsson Fisk, MD[1]
Martin Lindström MD, PhD, Prof[2,3]
Raquel Perez-Vicente. MSc[1]
Juan Merlo, MD, PhD, Prof[1,3]

[1] Unit for Social Epidemiology, Department of Clinical Sciences, Lund University, Malmö, Sweden
[2] Unit for Social Medicine and Health Policy, Department of Clinical Sciences, Lund University, Malmö, Sweden
[3] Centre for Primary Health Care Research, Region Skåne, Malmö, Sweden

Corresponding author
Sten Axelsson Fisk
Unit for Social Epidemiology, Lund University
Jan Waldenströms gata 35
214 28 Malmö
Sweden
Phone: +46-701-732506
Email: sten.axelsson_fisk@med.lu.se

Words: 3968 (excluding abstract, article summary, abbreviations and references)
Tables: 3
Figures: 2
Supplemental material: 4

For peer review only

Abstract (254 words)

Objectives: Socioeconomic disparities in smoking prevalence remains a challenge to public health. The objective of this study was to present a simple methodology that displays intersectional patterns of smoking and quantify heterogeneities within groups to avoid inappropriate and potentially stigmatizing conclusions exclusively based on group averages.

Setting: This is a cross-sectional observational study based on data from the National Health Surveys for Sweden (2004-2016 and 2018) including 136 301 individuals. We excluded people under 30 years of age, or missing information on education, household composition or smoking habits. The final sample consisted on 110 044 individuals or 80.7% of the original sample.

Outcome: Applying intersectional analysis of individual heterogeneity and discriminatory accuracy (AIHDA), we investigated the risk of self-reported smoking across 72 intersectional strata defined by age, gender, educational achievement, migration status and household composition.

Results: The distribution of smoking habit risk in the population was very heterogeneous. For instance, immigrant men aged 30–44 with low educational achievement that lived alone had a prevalence of smoking of 54% (95%CI 44–64%), around 9 times higher than native women aged 65–84 with high educational achievement and living with other(s) that had a prevalence of 6% (95%CI 5–7%). The discriminatory accuracy of the information was moderate.

Conclusion: A more detailed, intersectional mapping of the socioeconomic and demographic disparities of smoking can assist in public health management aiming to eliminate this unhealthy habit from the community. Intersectionality theory together with AIHDA provides information that can guide resource allocation according to the concept proportionate universalism.

For peer review only

Strengths and limitations

- We present an intersectional approach to study the multidimensional socioeconomic disparities in smoking prevalence in Sweden.
- In addition to differences between averages of intersectional strata, we quantify individual heterogeneities around those averages by presenting measurements of discriminatory accuracy.
- Our method is simpler but share crucial advantages with Multilevel AIHDA, such as improved health mapping and assessment of intersectional interaction.
- We use pooled data from Swedish National Health Survey with participation rates spanning from 60.8% 2004 to 42.1% 2018.
- Analysis of Individual Heterogeneity and Discriminatory Accuracy (AIHDA) is a suitable tool to inform whether interventions to reduce socioeconomic health disparities should be universal or target specific groups.

For peer review only - http://bmjopen.bmj.com/site/about/guidelines.xhtml

Abbreviations

AIHDA=Analysis of Individual Heterogeneity and Discriminatory Accuracy, AUC=Area Under receiving operator characteristics Curve, CI=Confidence Interval, DA=Discriminatory Accuracy, OR=Odds Ratio, PR=Prevalence Ratio, SDH=Social Determinants of Health

For peer review only

For peer review only - http://bmjopen.bmj.com/site/about/guidelines.xhtml

Introduction

A higher prevalence of smoking among individuals with low socioeconomic position (SEP) compared to higher SEP has been reported in several studies in Sweden [1] and globally [2-5]. The higher prevalence results both from higher rates of initiation [6] and lower rates of successful smoking cessation [7]. In addition to this, other factors like country of birth [8], household composition [9], age and gender influence the probability of smoking [10]. Overall, socioeconomic determinants of smoking are multidimensional but few studies have empirically confronted this heterogeneity using an intersectional perspective [11-15].

Intersectionality theory, proportionate universalism, and the analysis of individual heterogeneity and discriminatory accuracy (AIHDA)

Structural interventions including raised tobacco taxes and smoking free zones can reduce smoking prevalence [16], most among people with low SEP [17]. In UK, health-care based smoking cessation aid has reduced disparities in smoking rates between privileged and socioeconomically deprived areas, although this effect was modest [18]. However, a review of the efficacy of non-health care interventions targeting behavioural factors among people with low education [19] concludes that there is a lack of evidence that such interventions oriented towards individual determinants of health are efficient when it comes to reducing socioeconomic disparities in smoking [20]. Marmot and Bell claim [21] that interventions to reduce socioeconomic health disparities need to address all levels of society and not only those who are worst off. They argue that an efficient approach may be *proportionate universalism* [21, 22] where interventions are universal, i.e. directed towards the whole population (such as tobacco taxes, smoking bans in public) but proportionately more intense among population subgroups with augmented needs where targeted interventions can be launched (i.e. information campaigns in specific neighbourhoods or populations such as pregnant women). However, as argued elsewhere [22-24] successful and efficient

For peer review only - http://bmjopen.bmj.com/site/about/guidelines.xhtml

implementation of *proportionate universalism* requires development and application of appropriate theories and epidemiologic methodologies.

Intersectionality theory is a critical social theory [25] that stresses the need for simultaneous consideration of different social dimensions such as racialized identity, gender and class in order to properly understand the social context acting on individuals. According to intersectionality theory, the social reality is shaped by overlapping systems of oppression that influence distribution of resources and power in society.

The inclusion of intersectionality in epidemiology and public health has been promoted by several scholars [26-29]. A direct consequence of this approach in quantitative analyses is the study of multiple intersectional strata defined by combinations of different social dimensions, since the effect of each social dimension on an individual is intrinsically dependent on other social identities of that person. This contrasts with the common approach considering one social dimension at the time. Thereby, the intersectional approach may enrich public health research by providing an improved mapping of socioeconomic health disparities [26, 30]. Such socioeconomic heterogeneity can be analyzed by quantifying differences between intersectional strata averages. However, we [23, 28, 29, 31, 32] and other scholars [33-35] stress the added relevance of simultaneously quantifying the discriminatory accuracy (DA) of the intersectional categorization for specific outcomes. An intersectional map combined with information on its DA provides an improved picture of the socioeconomic heterogeneity existing in the society. This approach can be used to inform interventions according to the concept of proportionate universalism. The extent to which a universal intervention needs to be proportional can be evaluated by the DA of the intersectional strata. A low DA suggests the need for universal interventions while a high DA supports more selective interventions. This idea aligns with the distinctions made by McCall between anti-, and inter-categorical

For peer review only - http://bmjopen.bmj.com/site/about/guidelines.xhtml

intersectional approaches [36]. According to the anti-categorical intersectionality, the categorizations adopted in quantitative research are simplified and contribute to stereotypes and perpetuations of inequalities. The inter-categorical intersectionality, on the other hand, accepts categorizations since they can be useful in the study of intersectional inequities. The finding of a low DA would support the anti-categorical standpoint that the categorizations lack relevance for the studied outcome. If the DA is high, this would rather support the inter-categorical standpoint that intersectional matrix provides worthy information. A moderate DA does not give full support to neither the anti- nor inter-categorical intersectionality.

Adopting a quantitative perspective, in the present study we aim to illustrate how a more precise intersectional categorization combined with AIHDA improves our understanding of smoking prevalence and facilitates the application of proportionate universalism.

Methods

Study population

In this cross-sectional observational study, we used data from all the 14 National Health Surveys (NHS) for Sweden for the years 2004–2016 and 2018 (https://www.folkhalsomyndigheten.se/the-public-health-agency-of-sweden/public-health-reporting/). The NHS is an ongoing collaborative project between the Public Health Agency of Sweden and the Swedish Association of Local Authorities and Regions. The NHS record self-reported information on health, lifestyle and living conditions. The study has been conducted annually between 2004 and 2016 and comprised a random sample of 20,000 individuals aged 16–84 years. After 2016 the survey is conducted biannually but with a random sample of 40,000 individuals. Response rates span from 60.8% 2004 to 42.1% 2018. Using a unique personal identification number, the Swedish authorities linked the sample

For peer review only - http://bmjopen.bmj.com/site/about/guidelines.xhtml

surveys to national register administered at Statistics Sweden to obtain demographical and socioeconomic information.

For our study we pooled the data from the last 14 surveys, which rendered a sample of 136 301 individuals. Thereafter, we excluded people younger than 30 years. The lower age limit of 30 years was chosen since most individuals in Sweden that will complete a three-year education after high school do so before this age [37] and educational status was the indicator of socioeconomic position chosen in this study. We also excluded people with missing information on education, household composition or smoking habits. The final sample consisted on 110 044 individuals or 80.7% of the original sample (Figure 1).

The present investigation was approved by the Swedish Ethical Review Authority (Dnr: 2019-01793) and the data safety committee at the Public Health Agency of Sweden.

Patient and public involvement

All data from NHS provided to researchers is anonymized, so study participants cannot be identified. The study participants were not involved in the research process.

Assessment of variables

Smoking status was assessed based on the answer to the question "Do you smoke?", if the person answered "Yes" or "Yes, sometimes", the individual was categorized as a smoker, if the respondent answered "No" the individual was considered a non-smoker.

We categorized *age* into three groups: 30-44, 45-64 and 65-84-year-old. We classified gender as a binary variable distinguishing between men and women as more specific information on gender was not available in the questionnaire. We classified *educational achievement* into three categories, as low if the respondent had not completed three years of high school education, as middle if they had high school education but less than three years of education after high school and high if the respondent had at least three years of education after high

For peer review only - http://bmjopen.bmj.com/site/about/guidelines.xhtml

school. Throughout 2008-2016 respondents were asked "with whom do you share household?", we defined *household composition* as living alone if the respondent answered "with no one", otherwise as living with other(s). In 2018 that question was not asked so individuals were defined in the same way according to the linked information provided by Statics Sweden. We classified *migration status* as native (i.e., born in Sweden) or immigrant.

As a way of operationalising intersectional contexts, we created 72 strata by combining the three categories of age, the two of gender, the three of educational achievement, the two of migration status and the two categories of household composition. We used 30–45-year-old, native men living with other(s) and with high educational achievement as the reference in the comparisons, as this group was assumed to occupy the position of greatest structural privilege. This choice was based on unidimensional assumptions of structural privilege for young compared to old [38], men compared to women, high SEP compared to low SEP [39], natives compared to immigrants [40] and those living with other(s) compared to people living alone [41]. We also included the survey year of the participants using 2018 as reference in all comparisons.

Statistical analyses

The first step in our analysis was to obtain the trends in smoking prevalence and the trends in socioeconomic and demographic gradients in smoking between 2004 and 2018 (see supplementary information S1). Thereafter, we performed a stratified analysis aimed to provide a detailed map of the prevalence (i.e., absolute risk) and 95% confidence intervals (CI) of smoking across the intersectional strata. This stratification allows comparing the prevalence of smoking in different strata without any reference (Figure 2).

Thereafter, we performed seven consecutive regression analyses, modelling smoking as the dependent variable and survey year as well as the different demographical and socioeconomic

For peer review only - http://bmjopen.bmj.com/site/about/guidelines.xhtml

dimensions alone and in combination as explanatory variables. The use of logistic regression to obtain odds ratios (OR) is common but the OR is a good estimation of the relative risk only when the prevalence of the outcome is very small (rare event assumption) [42]. Therefore, for the analysis, rather than logistic regression to obtain ORs, we used Cox proportional hazards regression with a constant follow-up time equal to one to obtain prevalence ratios (PR) [43] with 95% CI.

Model 1 included only survey year, model 2 added age, model 3 added gender, model 4 added educational achievement, model 5 added migration status and model 6 added household composition and thus included all the variables that defined the intersectional strata. Finally, the intersectional model 7 included the same variables as model 6 but in the form of a multicategorical variable with 72 intersectional strata. Here, we used the 30–45-year-old, native men living with other(s) and with high educational achievement as the reference in the comparison.

For each model, we quantified its DA by means of the area under the receiver operator characteristics curve (AUC) [44]. The AUC measures the accuracy of the information provided by the variables in the model for discriminating individuals who smoke from those who don't. The AUC takes a value between 0.5 and 1, where 1 indicates perfect discrimination and 0.5 means that the studied variables have no DA at all. The AUC can even be used to qualify the size of the intersectional differences. Rather than evaluating the absolute risk differences between strata, using the AUC we assess the overlapping of the individual risk predictions (based on the intersectional strata) between smokers and non-smokers.

There is no fully established practical guideline for the interpretation of the size of the AUC as a measure of DA when analysing intersectional inequalities. However, based on the cut-off values provided by Hosmer and Lemeshow [45] but using more neutral denominations we

For peer review only - http://bmjopen.bmj.com/site/about/guidelines.xhtml

qualify intersectional inequalities according to the DA as (i) “absent or very small” (AUC= 0.5–0.6), (ii) “moderate” (AUC >0.6–≤ 0.7), (iii) “large” (AUC >0.7– ≤ 0.8) and (iv) “very large” (AUC >0.8). Evaluating intersectional differences using only strata prevalence is insufficient as it does not consider any overlapping between the strata. Therefore, the AUC provides fundamental information for evaluation of group differences [46].

We further calculated the incremental change in the AUC value (Δ-AUC) between the models. The Δ-AUC quantifies the improvement in the DA obtained by a model, in relation to the previous model [24]. The categorical intersectional variable in model 7 allows for the capturing of interaction of effects. If any such interaction exists, the DA of model 7 will increase in comparison with model 6 and the Δ-AUC will thus be positive.

We used STATA version 15.1 and IBM SPSS (Statistical Package for the Social Sciences) version 25 for PC to perform all statistical analyses.

Results

Over the whole study period, the prevalence of smoking was 18%. The visual analysis of the trends indicated that the prevalence of smoking monotonically decreased in Sweden from 25.0% in 2004 to around 11.1% in 2018. While sex-differences were small throughout the period and the sex-category with highest smoking prevalence changed, we observed consistent differences between groups defined by age, country of birth, educational achievement and household composition. In absolute terms, the gaps between subgroups were static except for differences between age categories that narrowed in later years (see supplementary information S1).

Table 1 presents the prevalence of smokers and non-smokers across the included socioeconomic and demographic variables as well as across survey years. It indicates that the prevalence of smoking was higher in individuals aged 45-64 years (20.6%) than in both

For peer review only - http://bmjopen.bmj.com/site/about/guidelines.xhtml

younger (19.8%) and older people (12.4%). Women and men had similar prevalence of smoking (17.9% vs 17.8%). As expected, smoking was more common among people with low (21.7%) and medium (17.0%) educational achievement compared to people with high educational achievement (11.9%). The prevalence of smoking was higher among immigrants (23.9%) than among natives (17.0%) and the same was true for individuals living alone (24.1%) compared to those who were living with other(s) (16.5%).

Figure 2 shows the prevalence of smoking across the intersectional strata. We observed the highest prevalence (54%) among 30-44-year-old immigrant men with low educational achievement and living alone, and the lowest prevalence (6%) among 65-84-year-old native women with high educational achievement and living together. The reference stratum (i.e., 30–45-year-old, native men living with other(s) and with high educational achievement) used in the relative comparisons (Table 2) presented a smoking prevalence of about 12%.

The table 3 informs that the PR of smoking decreases with age, being lowest in the old population. This age gradient is clear after adjustment for the other variables in the model 6. Low educational achievement, being immigrant and living alone was associated with a higher smoking risk. However, there were no age-adjusted gender differences. The AUC in the model including only survey year was 0.58. In the age adjusted model 2, the AUC was 0.60 and it did not increase when gender was included in model 3. The AUC increased by 0.04 units when including education. It did not increase when adding migration status but further increased by 0.01 units when including household composition. The AUC of intersectional model 7 was 0.66, with 95%CI overlapping the AUC of model 6 indicating no conclusive intersectional interaction.

Table 2 shows the 10 strata with the lowest and the 10 strata with the highest PRs of smoking using the strata of young native men with high educational achievement and living with

For peer review only - http://bmjopen.bmj.com/site/about/guidelines.xhtml

other(s) as reference. The lowest PR= 0.55 was observed in older native women with high educational achievement and living with other(s) and the highest PR= 4.45 was observed in young immigrant men with low educational achievement and living alone. When comparing with the reference stratum of native young men with high educational achievement and living with other(s), we observed that low educational achievement, being immigrant and living alone were respectively present in seven, eight and nine of the ten strata with the highest risk of smoking (see the Supplementary information S2 for the complete list of PR-values).

Discussion

Main findings

Our study provides an improved mapping of the distribution of the smoking habit in Sweden compared to unidimensional analyses. Rather than focusing on single socioeconomic and demographical variables, we use an intersectional AIHDA analysis that uncovers the socioeconomic and demographical heterogeneity existing in the country. We also applied the AUC to obtain information on the accuracy of the intersectional grouping for identifying individuals according to their smoking status. We found a moderate AUC= 0.66, which indicates that individual risk of smoking considerably overlaps between the intersectional strata and that neither the anti-categorical nor the inter-categorical intersectionality approaches are fully supported. We found that the stratum-specific risks were due to the main effects of the different variables used to define the intersectional strata without any conclusive interactive component.

We found intersectional strata with a rather high prevalence of smoking. For instance, the prevalence of smoking in young immigrant men with low educational achievement and living alone was 54%. Interestingly, while high educational achievement generally prevents

For peer review only - http://bmjopen.bmj.com/site/about/guidelines.xhtml

smoking, young immigrant women that lived alone had a PR of 2.87 (1.86-4.42) despite their high educational achievement. This indicates that the protective effect of high education may depend on other variables such as migration status and gender. Our finding could hypothetically reflect both smoking culture in the country of birth of the individual or that discrimination on the basis of gender or migration status may contribute to making education a poorer indicator of socioeconomic position in this group.

Relation to previous studies

In spite of the use of different definitions and measurements of smoking habits as well as the use of different indicators of socioeconomic position, many previous publications have shown the existence of socioeconomic, ethnic and demographical differences in smoking [2, 4, 47]. However, as far we known, only a few have considered the intersectional approach [11, 14, 15]. The heterogeneous distribution of smoking prevalence we found in Sweden is in accordance with recent intersectional research on smoking cessation in the U.S. adult population [15].

High education may influence smoking through both direct effects, such as increased understanding of detrimental health effects of smoking, and indirect effects such as social and material circumstances [48]. Educational achievement is the preferred indicator of socioeconomic position in previous public health reports in Sweden [49]. We performed a sensitivity analysis where we included income instead of education and the results were very similar and are provided as supplementary material (S3).

In a comparison of the relative importance of low education on smoking prevalence across age- and gender groups in Denmark and Sweden, Eek et al. found that the effect of low education on smoking prevalence and continuation of smoking was strongest among younger

For peer review only - http://bmjopen.bmj.com/site/about/guidelines.xhtml

women in Sweden, indicating a failure of tobacco prevention interventions to reach this group [1]. While immigrant men were clearly overrepresented among the strata with highest prevalence of smoking, this was not the case for women. This pattern was also found by Lindström et al in a study from southern Sweden showing lower rates of smoking among men born in Sweden, but higher rates of smoking among women born in Sweden compared to men and women from most other country groups [8]. These differences were attributable to different smoking prevalence in the countries of origins of the immigrants, potentially representing different stages of the smoking transition. The distribution of smoking prevalence across age groups we found is similar to the pattern observed by Ali et al in a study from southern Sweden [50].

Strengths and limitations

The cross-sectional and observational character of this study prevents causal conclusions. However, the variables included in our analyses are to a little extent effected by smoking status, so the causal direction can be presumed to go from sociodemographic variables towards smoking rather than the opposite.

A weakness in our study is that the participation rates were rather low, especially during the last years. An analyses of the non-participants performed by Statistics Sweden shows that people with low income, people born outside Sweden and people living alone were less likely to be responders [51]. Therefore, if the prevalence of smoking is higher in non-participants, our analysis may have underestimated the existing socioeconomic differences. In a sensitivity analysis we utilized data that had been weighted by Statistics Sweden in order to reduce skewness resulting from non-participating individuals. The variables used to perform the weighting were age, gender, educational level, country of birth, household composition and urban/rural [52]. These results were very similar, which was expected since the intersectional

For peer review only - http://bmjopen.bmj.com/site/about/guidelines.xhtml

variable included all weighting variables except rural/urban. Our study represents the Swedish circumstances so the AIHDA-approach should be replicated in different contexts.

A further limitation of this study is the simple categorizations of the dimensions incorporated in the intersectional matrix. Gender was binary defined which neglects the existence of numerous gender identities. Migration status was binary defined as natives and immigrants, which may hide heterogeneity in smoking prevalence. A more detailed classification with four categories (i.e., Sweden, Nordic countries, Europe and Outside Europe) shows that all the categories except women born outside Europe had a higher prevalence than the individuals born in Sweden (see supplementary material 4). The used categorizations stem in part from the information available in the survey and in part from the aim of presenting a parsimonious intersectional model that is easier to adopt in public health analyses and by the fact that several strata would be empty or contain very few individuals if the intersectional matrix was expanded.

We also performed a sensitivity analysis excluding "sometimes smokers" from the smoker category. As expected, overall prevalence was lower, 11% compared to 18%, and intersectional disparities larger. The AUC of the intersectional model 7 was 0.70 compared to 0.66 in the main analysis. Our main results combined with the results from the sensitivity analysis reflect the existence of socioeconomic disparities not only in prevalence, but also in intensity, of smoking [53]. Our results therefore may underestimate the intersectional disparities in health hazards attributable to smoking.

Implications and future studies

There is a growing body of literature focusing on how to perform quantitative intersectional research [27, 36], with the emergence of multilevel AIHDA (MAIHDA) as a recent example

For peer review only - http://bmjopen.bmj.com/site/about/guidelines.xhtml

[28, 29, 34]. However, in spite of providing complementary information [34], the fixed effects AIHDA approach we use in our study is rather accessible and share crucial advantages of the MAIHDA. First, the AIHDA provides an intersectional mapping that is more appropriate than unidimensional analyses to identify specifically vulnerable population groups in which interventions could be effective. Second, by going beyond average probabilistic measurements (i.e., prevalence) and also analysing DA we get a quantification of the heterogeneity around the averages [46]. From the AIHDA we found that the DA of our intersectional model was only moderate which indicates the necessity for universal interventions due to a large unexplained heterogeneity. However, we also identified that the three most vulnerable groups (i.e., strata) included immigrant men with low education younger than 65 years. This finding suggest that special preventive measures should be directed to these groups. Furthermore, research methods that actively involves members of marginalized groups and has the explicit purpose to result in public health improvements are developing and could be one way forward [54].

Interventions to reduce smoking prevalence should address Social Determinants of Health (SDH) at all levels. Examples targeted directly at smoking include increased tobacco taxation, smoke free zones and public anti-smoking campaigns [55]. Stigmatization is a negative side effect of such interventions that need to be taken into account, especially for low SEP groups [56]. Qualitative intersectional research has provided important insights into how the stigma of smoking interacts with identities of low class, country of birth, being a bad mother and may be in conflict with norms of femininity [57].

Equal access to education, housing and healthy recreation, regardless of gender, socioeconomic status, migration status and household composition, is important to reduce smoking prevalence. Therefore, institutions outside the health care system play an important

For peer review only - http://bmjopen.bmj.com/site/about/guidelines.xhtml

role to redistribute resources and access to SDH [58, 59], in order to counterweight the accelerating tendency of accumulation of resources among a very rich minority that characterizes modern capitalism [60]. This requires political decisions that prioritize population health aims more than market oriented reforms that exacerbate health inequities [61]. Health politics should adopt an intersectional perspective when redistributing resources in order to reduce the complex disparities in smoking revealed in this study.

Conclusions

Compared with studies focused on single variables, the intersectional AIHDA offers a better mapping of the socioeconomic and demographical distribution of smoking in Sweden. However, the moderate DA found in the AIHDA analysis suggested the existence of substantial unexplained heterogeneity in smoking risk within the different intersectional strata defined by age, gender, education, household composition and migration status. An intersectional AIHDA approach is necessary to understand the existing socioeconomic and demographic complexity influencing smoking behaviour. Future studies should identify preventive measures that are guided by proportionate universalism to find practical ways forwards to reduce intersectional disparities in smoking prevalence.

Footnotes

Funding

This work was supported by grants to Juan Merlo (PI) from the Swedish Research Council (Vetenskapsrådet) for the project *"Multilevel Analyses of Individual Heterogeneity: innovative concepts and methodological approaches in Public Health and Social Epidemiology"* (https://www.swecris.se/betasearch/details/project/201701321VR?lang=en.

For peer review only - http://bmjopen.bmj.com/site/about/guidelines.xhtml

Project id: 2017-01321.) The study was also supported by a grant for advanced doctoral studies from the Faculty of Medicine at Lund University (Dnr 2020/1125, SAF)

The funders had no role in study design, data collection and analysis, decision to publish, or preparation of the manuscript.

We acknowledge the staff and participants of all the national health surveys that has made this study possible.

Contributors

SAF and JM had the original idea of the study. SAF wrote the initial version of the paper, SAF, JM, RPV and ML participated in the design, analysis, interpretation of data and drafting of the article. All authors approved both the original and revised versions to be published. JM is the guarantor of the article.

Conflict of interest

There are no conflicts of interest.

Data sharing statement

The original databases from the Public Health Agency of Sweden used in this study are protected by strict rules of confidentiality that cover sensitive individual information including data regarding health and health behaviours. The data can be made available for research after review by a regional Ethics Committee and the Public Health Agency's own safety committee.

Ethics approval

The present investigation was approved by the Swedish Ethical Review Authority (Dnr: 2019-01793) and the data safety committee at the Public Health Agency of Sweden.

For peer review only - http://bmjopen.bmj.com/site/about/guidelines.xhtml

License statement

I, the Submitting Author has the right to grant and does grant on behalf of all authors of the Work (as defined in the below author licence), an exclusive licence and/or a non-exclusive licence for contributions from authors who are: i) UK Crown employees; ii) where BMJ has agreed a CC-BY licence shall apply, and/or iii) in accordance with the terms applicable for US Federal Government officers or employees acting as part of their official duties; on a worldwide, perpetual, irrevocable, royalty-free basis to BMJ Publishing Group Ltd ("BMJ") its licensees and where the relevant Journal is co-owned by BMJ to the co-owners of the Journal, to publish the Work in any BMJ products and to exploit all rights, as set out in our licence.

For peer review only

References

[1] Eek F, Ostergren P-O, Diderichsen F*, et al.* Differences in socioeconomic and gender inequalities in tobacco smoking in Denmark and Sweden; a cross sectional comparison of the equity effect of different public health policies. *BMC public health* 2010;**10**:9-9.

[2] Kuntz B, Lampert T. Educational differences in smoking among adolescents in Germany: what is the role of parental and adolescent education levels and intergenerational educational mobility? *International journal of environmental research and public health* 2013;**10**(7):3015-3032.

[3] Härkönen J, Lindberg M, Karlsson L*, et al.* Education is the strongest socio-economic predictor of smoking in pregnancy. *Addiction* 2018;**113**(6):1117-1126.

[4] Hiscock R, Bauld L, Amos A*, et al.* Socioeconomic status and smoking: a review. *Annals of the New York Academy of Sciences* 2012;**1248**(1):107-123.

[5] Graham H. Gender and class as dimensions of smoking behaviour in Britain: insights from a survey of mothers. *Social science & medicine (1982)* 1994;**38**(5):691-698.

[6] Joffer J, Burell G, Bergstrom E*, et al.* Predictors of smoking among Swedish adolescents. *BMC public health* 2014;**14**:1296.

[7] Gilman SE, Martin LT, Abrams DB*, et al.* Educational attainment and cigarette smoking: a causal association? *International journal of epidemiology* 2008;**37**(3):615-624.

[8] Lindstrom M, Sundquist J. Ethnic differences in daily smoking in Malmo, Sweden. Varying influence of psychosocial and economic factors. *European journal of public health* 2002;**12**(4):287-294.

[9] Lindstrom M, Giordano GN. Changes in Social Capital and Cigarette Smoking Behavior Over Time: A Population-Based Panel Study of Temporal Relationships. *Nicotine & tobacco research : official journal of the Society for Research on Nicotine and Tobacco* 2016;**18**(11):2106-2114.

[10] Ramstrom L, Borland R, Wikmans T. Patterns of Smoking and Snus Use in Sweden: Implications for Public Health. *Int J Environ Res Public Health* 2016;**13**(11).

[11] Amroussia N, Pearson JL, Gustafsson PE. What drives us apart? Decomposing intersectional inequalities in cigarette smoking by education and sexual orientation among U.S. adults. *International journal for equity in health* 2019;**18**(1):109.

[12] Mena E, Bolte G, Group AGS. Intersectionality-based quantitative health research and sex/gender sensitivity: a scoping review. *International journal for equity in health* 2019;**18**(1):199-199.

[13] Bilal U, Beltrán P, Fernández E*, et al.* Gender equality and smoking: a theory-driven approach to smoking gender differences in Spain. *Tobacco control* 2016;**25**(3):295-300.

[14] Aguirre CG, Bello MS, Andrabi N*, et al.* Gender, Ethnicity, and Their Intersectionality in the Prediction of Smoking Outcome Expectancies in Regular Cigarette Smokers. *Behavior modification* 2016;**40**(1-2):281-302.

[15] Potter LN, Lam CY, Cinciripini PM*, et al.* Intersectionality and Smoking Cessation: Exploring Various Approaches for Understanding Health Inequities. *Nicotine & Tobacco Research* 2020.

[16] Neuberger M. Tobacco control: prevention and cessation in Europe. *memo - Magazine of European Medical Oncology* 2019;**12**(2):156-161.

[17] Golden SD, Farrelly MC, Luke DA*, et al.* Comparing projected impacts of cigarette floor price and excise tax policies on socioeconomic disparities in smoking. *Tobacco control* 2016;**25**(Suppl 1):i60.

For peer review only - http://bmjopen.bmj.com/site/about/guidelines.xhtml

[18] Bauld L, Judge K, Platt S. Assessing the impact of smoking cessation services on reducing health inequalities in England: observational study. *Tobacco control* 2007;**16**(6):400-404.
[19] Vilhelmsson A, Ostergren PO. Reducing health inequalities with interventions targeting behavioral factors among individuals with low levels of education - A rapid review. *PloS one* 2018;**13**(4):e0195774.
[20] Wendel-Vos GC, Dutman AE, Verschuren WM*, et al.* Lifestyle factors of a five-year community-intervention program: the Hartslag Limburg intervention. *American journal of preventive medicine* 2009;**37**(1):50-56.
[21] Marmot M, Bell R. Fair society, healthy lives. *Public health* 2012;**126 Suppl 1**:S4-s10.
[22] Carey G, Crammond B, De Leeuw E. Towards health equity: a framework for the application of proportionate universalism. *International journal for equity in health* 2015;**14**:81.
[23] Wemrell M, Bennet L, Merlo J. Understanding the complexity of socioeconomic disparities in type 2 diabetes risk: a study of 4.3 million people in Sweden. *MJ Open Diabetes Research and Care* 2019;**9;7:e000749.**
[24] Merlo J, Wagner P, Leckie G. A simple multilevel approach for analysing geographical inequalities in public health reports: The case of municipality differences in obesity. *Health & place* 2019;**58**:102145.
[25] K C. Demarginalizing the intersection of race and sex: a Black feminist critique of antidiscriminiation doctrine, feminist theory and antiracist politics. *University of Chicago Legal Forum* 1989(139).
[26] Bauer GR. Incorporating intersectionality theory into population health research methodology: challenges and the potential to advance health equity. *Soc Sci Med* 2014;**110**:10-17.
[27] Bowleg L. When Black plus Lesbian plus Woman not equal Black Lesbian Woman: The methodological challenges of qualitative and quantitative intersectionality research. *Sex Roles* 2008;**59**(5-6):312-325.
[28] Merlo J. Multilevel analysis of individual heterogeneity and discriminatory accuracy (MAIHDA) within an intersectional framework. *Social science & medicine (1982)* 2018;**203**:74-80.
[29] Wemrell M, Mulinari S, Merlo J. An intersectional approach to multilevel analysis of individual heterogeneity (MAIH) and discriminatory accuracy. *Social science & medicine (1982)* 2017;**178**:217-219.
[30] Green MA, Evans CR, Subramanian SV. Can intersectionality theory enrich population health research? *Social science & medicine (1982)* 2017;**178**:214-216.
[31] Axelsson Fisk S, Mulinari S, Wemrell M*, et al.* Chronic Obstructive Pulmonary Disease in Sweden: An intersectional multilevel analysis of individual heterogeneity and discriminatory accuracy. *SSM Popul Health* 2018;**4**:334-346.
[32] Persmark A, Wemrell M, Zettermark S*, et al.* Precision public health: Mapping socioeconomic disparities in opioid dispensations at Swedish pharmacies by Multilevel Analysis of Individual Heterogeneity and Discriminatory Accuracy (MAIHDA). *PLoS One* 2019;**14**(8):e0220322.
[33] Evans CR, Erickson N. Intersectionality and depression in adolescence and early adulthood: A MAIHDA analysis of the national longitudinal study of adolescent to adult health, 1995-2008. *Social science & medicine (1982)* 2019;**220**:1-11.
[34] Evans CR, Leckie G, Merlo J. Multilevel versus single-level regression for the analysis of multilevel information: The case of quantitative intersectional analysis. *Social science & medicine (1982)* 2019:112499.

For peer review only

For peer review only - http://bmjopen.bmj.com/site/about/guidelines.xhtml

[35] Evans CR, Williams DR, Onnela JP*, et al.* A multilevel approach to modeling health inequalities at the intersection of multiple social identities. *Soc Sci Med* 2017.
[36] McCall L. The complexity of intersectionality. *Signs: Journal of women in culture and society* 2005;**30**(3):1771-1800.
[37] OECD. Tertiary-type A graduation rates in 1995, 2000 and 2008 (first-time graduation) 2010.
[38] McMullin JA, Cairney J. Self-esteem and the intersection of age, class, and gender. *Journal of Aging Studies* 2004;**18**(1):75-90.
[39] Krieger N. A glossary for social epidemiology. *Journal of epidemiology and community health* 2001;**55**(10):693.
[40] Karlsen S, Nazroo JY. Measuring and analyzing "race," racism, and racial discrimination. *Methods in social epidemiology* 2017;**2**:43.
[41] Alm S, Nelson K, Nieuwenhuis R. The Diminishing Power of One? Welfare State Retrenchment and Rising Poverty of Single-Adult Households in Sweden 1988–2011. *European Sociological Review* 2019;**36**(2):198-217.
[42] Davies HT, Crombie IK, Tavakoli M. When can odds ratios mislead? *BMJ (Clinical research ed)* 1998;**316**(7136):989-991.
[43] Barros AJ, Hirakata VN. Alternatives for logistic regression in cross-sectional studies: an empirical comparison of models that directly estimate the prevalence ratio. *BMC Med Res Methodol* 2003;**3**:21.
[44] Pepe MS, Janes H, Longton G*, et al.* Limitations of the odds ratio in gauging the performance of a diagnostic, prognostic, or screening marker. *American journal of epidemiology* 2004;**159**(9):882-890.
[45] Hosmer DW, Lemeshow S. Applied logistic regression. 2nd ed. New York: Wiley 2000.
[46] Merlo J, Wagner P, Leckie G. A simple multilevel approach for analysing geographical inequalities in public health reports: The case of municipality differences in obesity. *Health Place* 2019;**58**:102145.
[47] Landberg J, Ramstedt M, Sundin E. Socioekonomiska skillnader i beroende och utsatthet för andras användning av alkohol, narkotika och tobak. Stockholm: Centralförbundet för alkohol- och narkotikaupplysning 2018.
[48] Östergren O. Understanding the educational gradient in Mortality: Department of Sociology, Stockholm University 2017.
[49] Folkhälsomyndigheten SkoR. Regional comparisons public health 2019. www.folkhalsomyndigheten.se/publicerat-material 2018.
[50] Ali SM, Chaix B, Merlo J*, et al.* Gender differences in daily smoking prevalence in different age strata: A population-based study in southern Sweden. *Scandinavian journal of public health* 2009;**37**(2):146-152.
[51] Franzén M. Extra bortfallsanalys. Statistics Sweden 2018.
[52] Franzén M. Kalibreringsrapport Nationellt Urval. Stockholm: Statistics Sweden 2015.
[53] Bobak M, Jarvis MJ, Skodova Z*, et al.* Smoke intake among smokers is higher in lower socioeconomic groups. *Tobacco control* 2000;**9**(3):310.
[54] Andrews JO, Newman SD, Heath J*, et al.* Community-based participatory research and smoking cessation interventions: a review of the evidence. *Nursing Clinics* 2012;**47**(1):81-96.
[55] Dahlgren G, Whitehead M. Policies and strategies to promote social equity in health. 1991.
[56] Graham H. Smoking, Stigma and Social Class. *Journal of Social Policy* 2011;**41**(1):83-99.

For peer review only - http://bmjopen.bmj.com/site/about/guidelines.xhtml

[57] Triandafilidis Z, Ussher JM, Perz J*, et al.* An Intersectional Analysis of Women's Experiences of Smoking-Related Stigma. *Qualitative Health Research* 2016;**27**(10):1445-1460.
[58] Gkiouleka A, Huijts T, Beckfield J*, et al.* Understanding the micro and macro politics of health: Inequalities, intersectionality & institutions-A research agenda. *Social Science & Medicine* 2018;**200**:92-98.
[59] Bambra C, Fox D, Scott-Samuel A. Towards a politics of health. *Health promotion international* 2005;**20**(2):187-193.
[60] Piketty T. About capital in the twenty-first century. *American Economic Review* 2015;**105**(5):48-53.
[61] Burström B. Market-Oriented, Demand-Driven Health Care Reforms and Equity in Health and Health Care Utilization in Sweden. *International Journal of Health Services* 2009;**39**(2):271-285.

For peer review only

For peer review only - http://bmjopen.bmj.com/site/about/guidelines.xhtml

Tables

Table 1. Distribution (prevalence) of smokers across categories of age, gender, education, migration and household composition in the 110,044 participants in the Swedish National Health Surveys (2004 – 2018). Values are number (and percentage) of individuals.

	Non smokers	Smokers
30-44	22,799 (80.23%)	5,618 (19.77%)
45-64	38,024 (79.41%)	9,862 (20.59%)
65-84	29,575 (87.65%)	4,166 (12.35%)
Female	48,782 (82.08%)	10.653 (17.92%)
Male	41,616 (82.23%)	8,993 (17.77%)
Low	38,791 (78.32%)	10,738 (21.68%)
Middle	27,716 (83.02%)	5,670 (16.98)
High	23,891 (88.06%)	3,238 (11.94%)
Immigrant	10,410 (76.07%)	3,274 (23.93%)
Native	79,988 (83.01%)	16,372 (16.99%)
Living with other(s)	75,625 (83.48%)	14,964 (16.52%)
Living Alone	14,773 (75.93%)	4,682 (24.07%)
2004	6,803 (75.03%)	2,264 (24.97%)
2005	3,339 (75.90%)	1,060 (24.10%)
2006	3,450 (77.62%)	995 (22.38%)
2007	3,272 (77.81%)	933 (22.19%)
2008	6,525 (79.07%)	1,727 (20.93%)
2009	6,123 (79.22%)	1,606 (20.78%)
2010	6,718 (80.59%)	1,618 (19.41%)
2011	6,760 (82.56%)	1,428 (17.44%)
2012	6,893 (82.68%)	1,444 (17.32%)
2013	6,770 (83.10%)	1,377 (16.90%)
2014	6,845 (83.74%)	1,329 (16.26%)
2015	6,978 (84.21%)	1,308 (15.79%)
2016	7,086 (88.13%)	954 (11.87%)
2018	12,836 (88.90%)	1,603 (11.10%)

For peer review only

For peer review only - http://bmjopen.bmj.com/site/about/guidelines.xhtml

Table 2. Results from the intersectional model 7 indicating the 10 highest and 10 lowest Prevalence Ratio (PR) with 95% confidence intervals (CI) of smoking across intersectional strata in the Swedish population using the stratum of young, native, men with high education that were living with other(s) (LWO) as reference in the comparisons.

Age	Gender	Educational achievement	Migration status	Household composition	PR (95% CI)
65-84	Female	High	Native	LWO	0.55 (0.45-0.69)
65-84	Male	High	Native	LWO	0.58 (0.48-0.71)
65-84	Female	High	Immigrant	LWO	0.61 (0.33-1.11)
65-84	Female	Middle	Native	LWO	0.80 (0.66-0.96)
65-84	Female	High	Native	Living alone	0.83 (0.64-1.06)
65-84	Male	Middle	Native	LWO	0.85 (0.73-0.99)
30-44	Female	High	Native	LWO	0.86 (0.74-0.98)
65-84	Male	High	Immigrant	Living alone	0.91 (0.38-2.21)
45-64	Male	High	Native	LWO	0.92 (0.8-1.07)
65-84	Male	Low	Native	LWO	0.96 (0.84-1.11)
30-44	**Male**	**High**	**Native**	**LWO**	**Reference**
30-44	Female	High	Immigrant	Living alone	2.87 (1.86-4.42)
30-44	Female	Low	Native	Living alone	2.95 (2.29-3.78)
45-64	Female	Low	Native	Living alone	2.99 (2.61-3.41)
45-64	Male	Middle	Immigrant	Living alone	3.10 (2.26-4.26)
45-64	Female	Low	Immigrant	Living alone	3.22 (2.56-4.06)
30-44	Male	Middle	Immigrant	Living alone	3.33 (2.35-4.71)
30-44	Female	Low	Immigrant	Living alone	3.41 (1.96-5.94)
45-64	Male	Low	Immigrant	Living alone	3.61 (2.90-4.50)
30-44	Male	Low	Immigrant	LWO	3.66 (3.07-4.35)
30-44	Male	Low	Immigrant	Living alone	4.45 (3.29-6.03)
AUC					0.66 (0.65-0.66)
ΔAUC compared with model 6					0.01

For peer review only

For peer review only - http://bmjopen.bmj.com/site/about/guidelines.xhtml

Table 3. Prevalence ratios (PR) and 95%-confidence intervals (CI), of smoking among people aged 30-84 included in the National Health Surveys between 2004 and 2018 in relation to survey year, age, gender, education, migration statis and household composition. Model 7 includes the same variables as model 6 but as a multicategorical variable, The PRs and AUC for model 7 are presented in the table 2. AUC-values with 95%CI representing the discriminatory accuracy and ΔAUC-values of the models are also presented.

		Model 1	Model 2	Model 3	Model 4	Model 5	Model 6
Year							
	2004	2.25 (2.11-2.40)	2.07 (1.95-2.21)	2.08 (1.95-2.21)	1.85 (1.74-1.98)	1.88 (1.76-2.00)	1.84 (1.73-1.97)
	2005	2.17 (2.01-2.35)	2.01 (1.86-2.17)	2.01 (1.86.2.17)	1.83 (1.69.1.98)	1.85 (1.71-2.00)	1.83 (1.69-1.98)
	2006	2.02 (1.86-2.18)	1.87 (1.72-2.02)	1.87 (1.72-2.02)	1.70 (1.57-1.84)	1.71 (1.58-1.85)	1.68 (1.55-1.82)
	2007	2.00 (1.84 -2.17)	1.86 (1.71.2.01)	1.86 (1.71-2.01)	1.71 (1.57-1.85)	1.65 (1.54-1.77)	1.70 (1.56.1.84)
	2008	1.89 (1.76-2.02)	1.76 (1.64-1.88)	1.76 (1.64-1.88)	1.64 (1.53-1.75)	1.66 (1.55-1.78)	1.63 (1,52-1.74)
	2009	1.87 (1.75-2.01)	1.75 (1.64-1.89)	1.76 (1.64-1-88)	1.65 (1.54-1.77)	1.62 (1.51-1.73)	1.63 (1.52-1.75)
	2010	1.75 (1.63-1.87)	1.70 (1.58-1.82)	1.70 (1.58-1.82)	1.61 (1.50-1.72)	1.46 (1.36-1-57)	1.59 (1.48-1.70)
	2011	1.57 (1.46-1.69)	1.53 (1.43-1.64)	1.53 (1.43-1-64)	1.45 (1.35-1.56)	1.47 (1.37-1.58)	1.43 (1.33-1.54)
	2012	1.56 (1.45-1.68)	1.53 (1.42-1.64)	1.53 (1.42-1.64)	1.47 (1.37-1.57)	1.45 (1.35-1.56)	1.44 (1.34-1.55)
	2013	1.52 (1-42-1.64)	1.49 (1.39-1.60)	1.49 (1.39-1.60)	1.45 (1.35-1.55)	1.45 (1.35-1.56)	1.42 (1.32-1.52)
	2014	1.47 (1.36-1.75)	1.45 (1.35-1.56)	1.45 (1.35-1.56)	1.41 (1.31-1.52)	1.42 (1.32-1.53)	1.39 (1.30-1.50)
	2015	1.42 (1.32-1.53)	1.40 (1.30-1.51)	1.40 (1.30-1.51)	1.37 (1.27-1.47)	1.38 (1.28-1.48)	1.35 (1.26-1.46)
	2016	1.07 (0.99-1.16)	1.06 (0.97-1.14)	1.06 (0.97-1.14)	1.04 (0.96-1.13)	1.05 (0.97-1.13)	1. 03 (0.95-1.11)
	2018	Reference	Reference	Reference	Reference	Reference	Reference
Age							
	30-44		Reference	Reference	Reference	Reference	Reference
	45-64		1.06 (1.03-1.10)	1.06 (1.03-1.10)	0.94 (0.91-0.97)	0.94 (0.91-0.98)	0.93 (0.90-0.96)
	65-84		0.68 (0.65-0.71)	0.68 (0.65-0.71)	0.56 (0.54-0.58)	0.57 (0.55-0.59)	0.53 (0.51-0.56)
Gender							
	Male			Reference	Reference	Reference	Reference
	Female			1.00 (0.97-1.02)	1.02 (0.99-1.05)	1.02 (0.99-1.05)	1.01 (0.98-1.04)
Education							
	Low				1.96 (1.88-2.04)	1.96 (1.88-2.04)	1.93 (1.86-2.01)
	Middle				1.42 (1.36-1.48)	1.43 (1.37-1.49)	1.42 (1.36-1.49)
	High				Reference	Reference	Reference

For peer review only

Born in Sweden							
	Native					Reference	Reference
	Immigrant					1.39 (1.34-1.45)	1.39 (1.24-1.44)
Living alone							
	Living alone						1.53 (1.48-1.58)
	living with other(s)						Reference
AUC		0.58 (0.58-0.59)	0.60 (0.60-0.61)	0.60 (0.60-0.61)	0.64 (0.63-0.64)	0.64 (0.64-0.65)	0.65 (0.65-0.66)
ΔAUC		-	0.02	0.00	0.04	0.00	0.01

For peer review only - http://bmjopen.bmj.com/site/about/guidelines.xhtml

Legend to the figures

Figure 1. Flow chart showing the selection of the study population.

Figure 2. Absolute risk (i.e., prevalence) and 95% confidence intervals of smoking in different intersectional strata according the National Health Survey in Sweden between 2004 and 2018.

For peer review only

For peer review only - http://bmjopen.bmj.com/site/about/guidelines.xhtml

Figure 1. Flow chart showing the selection of the study population.

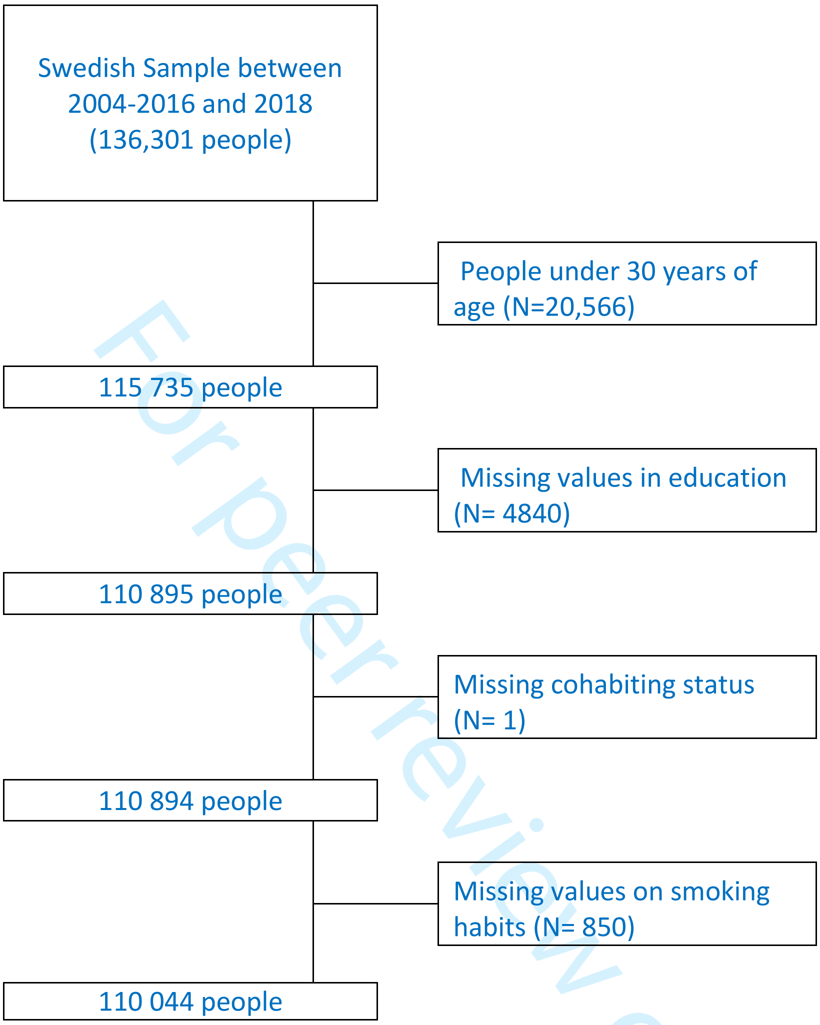

For peer review only

Figure 2. Absolute risk (i.e., prevalence) and 95% confidence intervals of smoking in different intersectional strata according the National Health Survey in Sweden between 2004 and 2018.

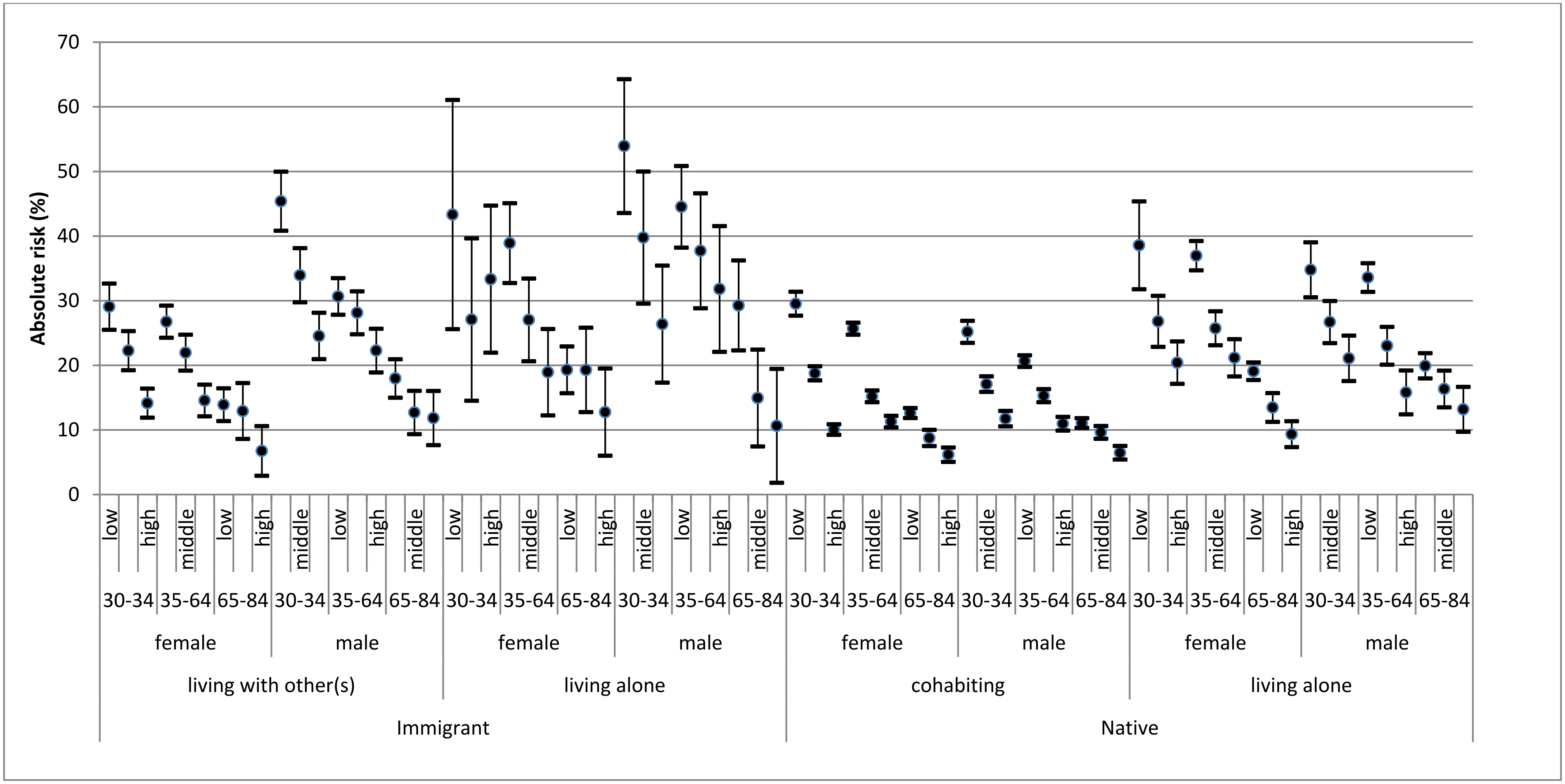

Supplementary material 1

S1.

Trends in smoking prevalence in the National Health Surveys for Sweden. Categories of age, gender, educational achievement, migration status and household composition are shown with different lines and 95% confidence intervals in the respective graphs.

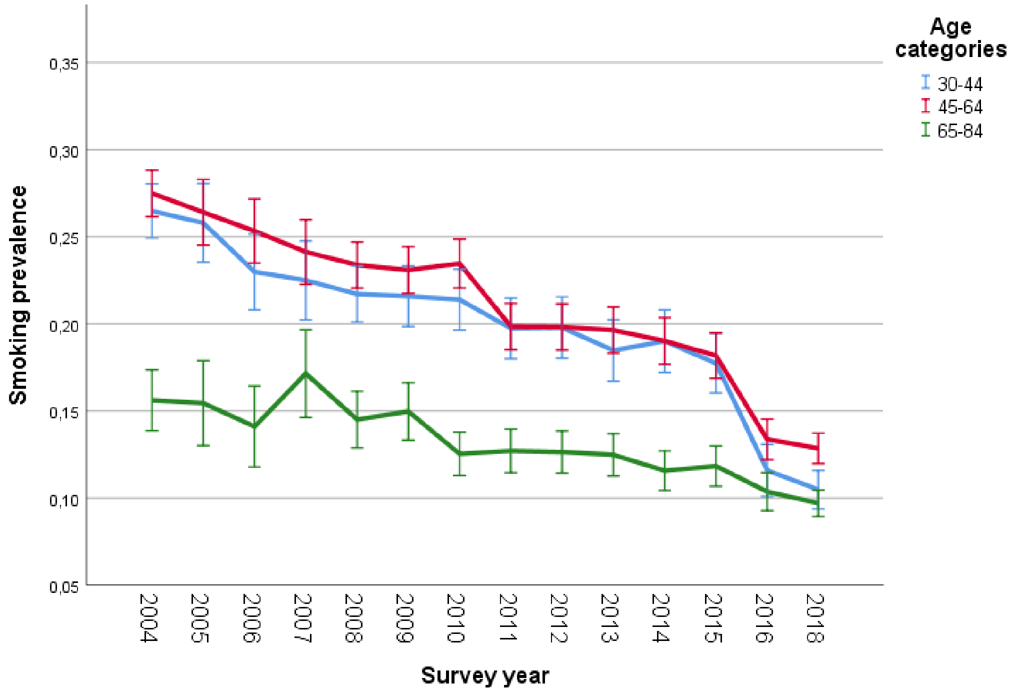

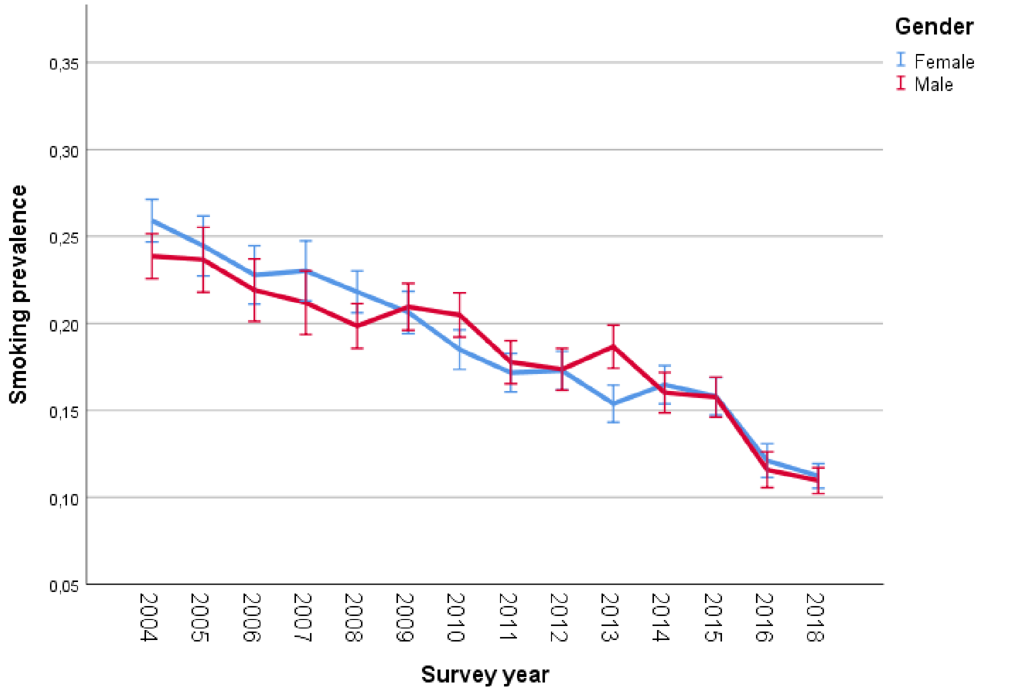

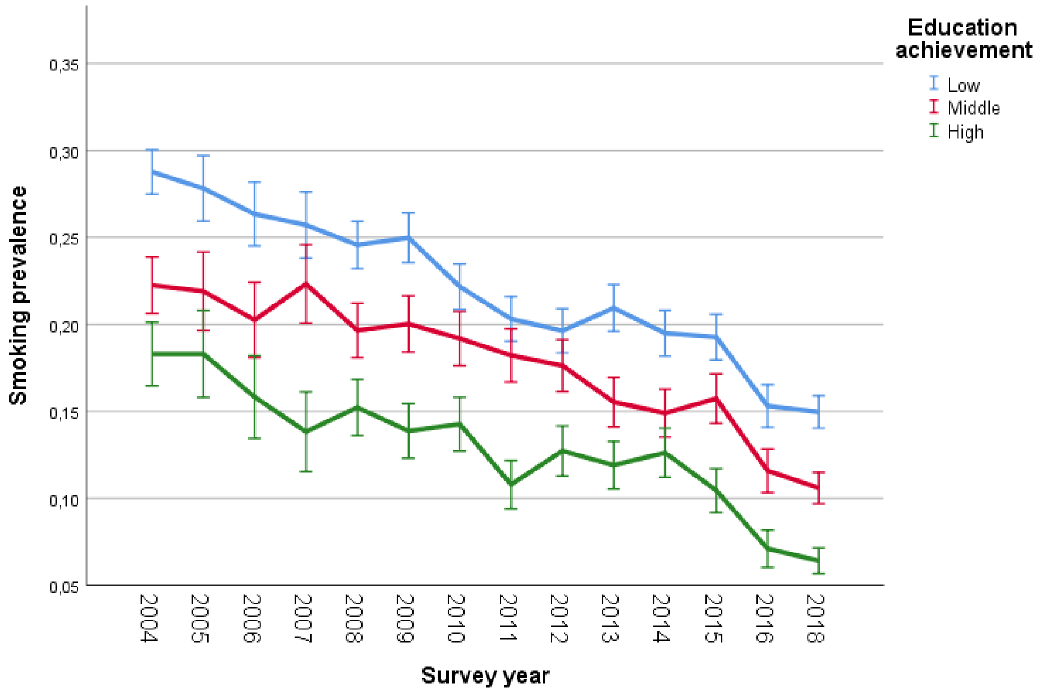
Education achievement
Low
Middle
High
Smoking prevalence
0,35
0,30
0,25
0,20
0,15
0,10
0,05
2004
2005
2006
2007
2008
2009
2010
2011
2012
2013
2014
2015
2016
2018
Survey year

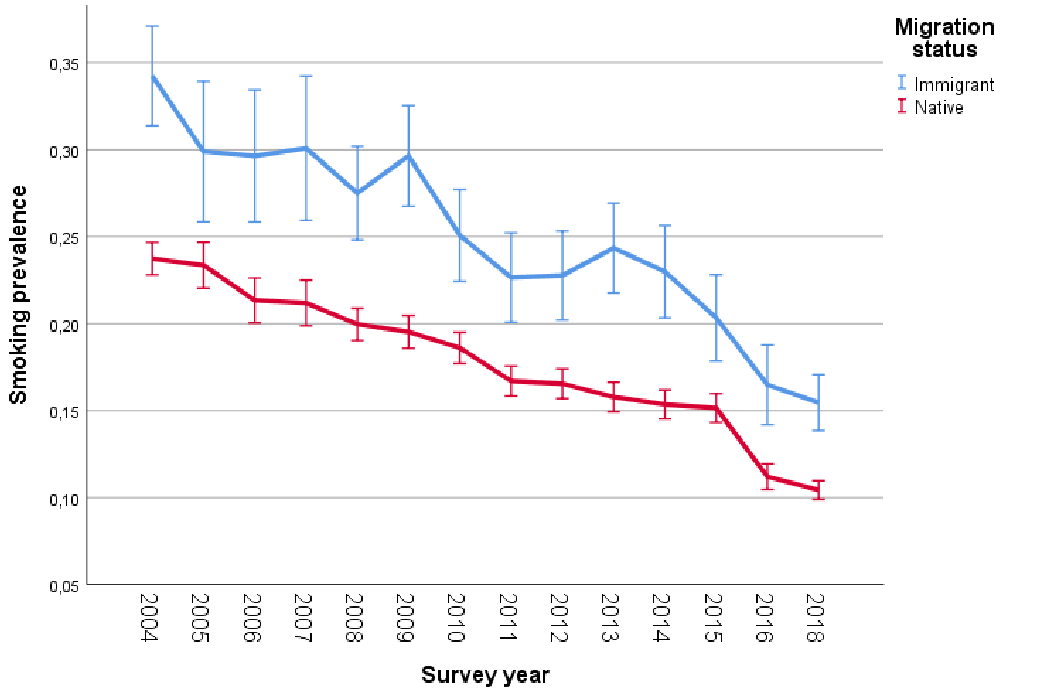
Migration status
Immigrant
Native
Smoking prevalence
0,35
0,30
0,25
0,20
0,15
0,10
0,05
2004
2005
2006
2007
2008
2009
2010
2011
2012
2013
2014
2015
2016
2018
Survey year

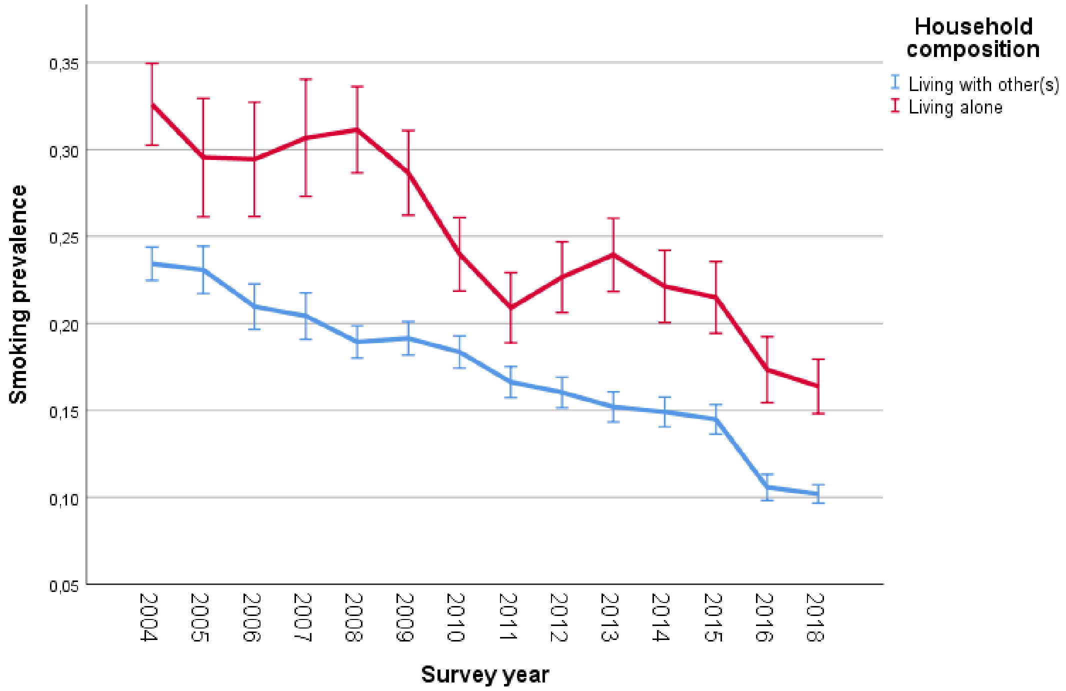
Household composition
Living with other(s)
Living alone
Smoking prevalence
0,35
0,30
0,25
0,20
0,15
0,10
0,05
2004
2005
2006
2007
2008
2009
2010
2011
2012
2013
2014
2015
2016
2018
Survey year

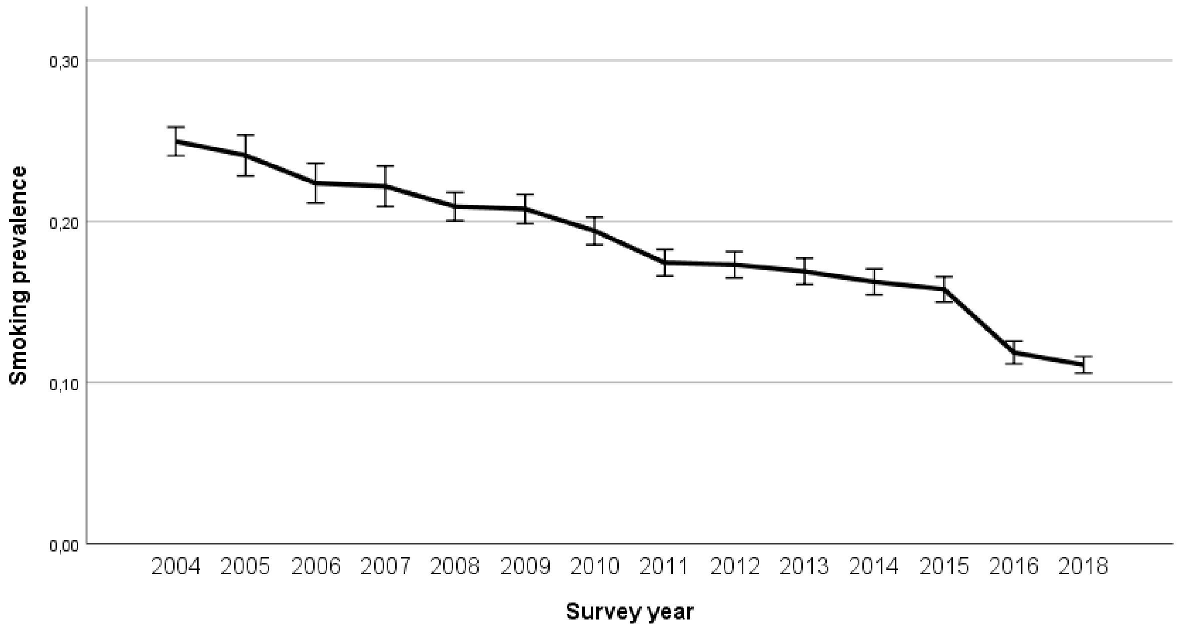
Smoking prevalence
0,30
0,20
0,10
0,00
2004
2005
2006
2007
2008
2009
2010
2011
2012
2013
2014
2015
2016
2018
Survey year

Supplementary material 2

S2

Table 2, full version. Results from the intersectional model 7 indicating the Prevalence Ratios (PR) with 95% confidence intervals (CI) of smoking across intersectional strata in the Swedish population using the stratum of young, native, men with high education that were living with other(s) (LWO) as reference in the comparisons.

Age	Gender	Educational achievement	Migration status	Household composition	PR (95% CI)
30-44	Female	Low	Immigrant	LWO	2.35 (1.96-2.82)
30-44	Female	Low	Immigrant	Living alone	3.41 (1.96-5.94)
30-44	Female	Low	Native	LWO	2.24 (1.96-2.56)
30-44	Female	Low	Native	Living alone	2.95 (2.29-3.78)
30-44	Female	Middle	Immigrant	LWO	1.83 (1.51-2.21)
30-44	Female	Middle	Immigrant	Living alone	2.33 (1.34-4.05)
30-44	Female	Middle	Native	LWO	1.53 (1.35-1.73)
30-44	Female	Middle	Native	Living alone	2.2 (1.8-2.7)
30-44	Female	High	Immigrant	LWO	1.22 (0.99-1.49)
30-44	Female	High	Immigrant	Living alone	2.87 (1.86-4.42)
30-44	Female	High	Native	LWO	0.86 (0.74-0.98)
30-44	Female	High	Native	Living alone	1.72 (1.39-2.12)
30-44	Male	Low	Immigrant	LWO	3.66 (3.07-4.35)
30-44	Male	Low	Immigrant	Living alone	4.45 (3.29-6.03)
30-44	Male	Low	Native	LWO	1.92 (1.68-2.2)
30-44	Male	Low	Native	Living alone	2.67 (2.21-3.21)
30-44	Male	Middle	Immigrant	LWO	2.84 (2.36-3.43)
30-44	Male	Middle	Immigrant	Living alone	3.33 (2.35-4.71)
30-44	Male	Middle	Native	LWO	1.43 (1.25-1.63)
30-44	Male	Middle	Native	Living alone	2.21 (1.85-2.64)
30-44	Male	High	Immigrant	LWO	2.13 (1.74-2.6)
30-44	Male	High	Immigrant	Living alone	2.32 (1.53-3.5)
30-44	Male	High	Native	LWO	Reference

For peer review only

30-44	Male	High	Native	Living alone	1.75 (1.41-2.18)
45-64	Female	Low	Immigrant	LWO	2.19 (1.88-2.55)
45-64	Female	Low	Immigrant	Living alone	3.22 (2.56-4.06)
45-64	Female	Low	Native	LWO	2.08 (1.85-2.34)
45-64	Female	Low	Native	Living alone	2.99 (2.61-3.41)
45-64	Female	Middle	Immigrant	LWO	1.87 (1.56-2.23)
45-64	Female	Middle	Immigrant	Living alone	2.23 (1.66-3.01)
45-64	Female	Middle	Native	LWO	1.29 (1.14-1.46)
45-64	Female	Middle	Native	Living alone	2.16 (1.84-2.53)
45-64	Female	High	Immigrant	LWO	1.27 (1.03-1.57)
45-64	Female	High	Immigrant	Living alone	1.63 (1.08-2.45)
45-64	Female	High	Native	LWO	0.97 (0.84-1.11)
45-64	Female	High	Native	Living alone	1.76 (1.46-2.13)
45-64	Male	Low	Immigrant	LWO	2.55 (2.18-2.98)
45-64	Male	Low	Immigrant	Living alone	3.61 (2.9-4.5)
45-64	Male	Low	Native	LWO	1.71 (1.52-1.93)
45-64	Male	Low	Native	Living alone	2.77 (2.42-3.17)
45-64	Male	Middle	Immigrant	LWO	2.39 (2.01-2.86)
45-64	Male	Middle	Immigrant	Living alone	3.1 (2.26-4.26)
45-64	Male	Middle	Native	LWO	1.28 (1.12-1.45)
45-64	Male	Middle	Native	Living alone	1.92 (1.61-2.31)
45-64	Male	High	Immigrant	LWO	1.91 (1.56-2.35)
45-64	Male	High	Immigrant	Living alone	2.7 (1.84-3.98)
45-64	Male	High	Native	LWO	0.92 (0.8-1.07)
45-64	Male	High	Native	Living alone	1.35 (1.04-1.75)
65-84	Female	Low	Immigrant	LWO	1.23 (0.98-1.54)
65-84	Female	Low	Immigrant	Living alone	1.62 (1.28-2.05)
65-84	Female	Low	Native	LWO	1.1 (0.97-1.25)
65-84	Female	Low	Native	Living alone	1.62 (1.42-1.86)

For peer review only

65-84	Female	Middle	Immigrant	LWO	1.18 (0.81-1.71)
65-84	Female	Middle	Immigrant	Living alone	1.66 (1.12-2.46)
65-84	Female	Middle	Native	LWO	0.8 (0.66-0.96)
65-84	Female	Middle	Native	Living alone	1.2 (0.97-1.48)
65-84	Female	High	Immigrant	LWO	0.61 (0.33-1.11)
65-84	Female	High	Immigrant	Living alone	1.16 (0.65-2.07)
65-84	Female	High	Native	LWO	0.55 (0.45-0.69)
65-84	Female	High	Native	Living alone	0.83 (0.64-1.06)
65-84	Male	Low	Immigrant	LWO	1.57 (1.27-1.95)
65-84	Male	Low	Immigrant	Living alone	2.49 (1.84-3.37)
65-84	Male	Low	Native	LWO	0.96 (0.84-1.1)
65-84	Male	Low	Native	Living alone	1.71 (1.47-2)
65-84	Male	Middle	Immigrant	LWO	1.12 (0.82-1.51)
65-84	Male	Middle	Immigrant	Living alone	1.29 (0.74-2.24)
65-84	Male	Middle	Native	LWO	0.85 (0.73-0.99)
65-84	Male	Middle	Native	Living alone	1.47 (1.18-1.83)
65-84	Male	High	Immigrant	LWO	1.06 (0.72-1.57)
65-84	Male	High	Immigrant	Living alone	0.91 (0.38-2.21)
65-84	Male	High	Native	LWO	0.58 (0.48-0.71)
65-84	Male	High	Native	Living alone	1.19 (0.88-1.6)

For peer review only

Supplementary material 3

S3

Sensitivity analysis where income tertiles were used as indicators of socioeconomic position instead of educational achievement.

Table 1. Results from the intersectional model 7 indicating the 10 highest and 10 lowest Prevalence Ratio (PR) with 95% confidence intervals (CI) of smoking across intersectional strata in the Swedish population using the stratum of young, native, men with high income that were living with other(s) (LWO) as reference in the comparisons.

Age	Gender	Income	Migration status	Civil status	RR (95% CI)
65-84	Male	High	Immigrant	LWO	0.51 (0.07-3.61)
65-84	Female	High	Immigrant	Living alone	0.64 (0.37-1.08)
65-84	Male	High	Native	Living alone	0.64 (0.55-0.74)
65-84	Female	High	Native	Living alone	0.66 (0.56-0.78)
65-84	Female	High	Native	LWO	0.71 (0.45-1.12)
65-84	Male	Middle	Native	Living alone	0.74 (0.65-0.83)
65-84	Female	Middle	Native	Living alone	0.76 (0.67-0.86)
65-84	Male	High	Immigrant	Living alone	0.77 (0.51-1.15)
65-84	Male	Low	Native	Living alone	0.87 (0.76-0.99)
65-84	Male	High	Native	LWO	0.91 (0.57-1.46)
30-44	**Male**	**High**	**Native**	**LWO**	**Reference**
30-44	Male	High	Immigrant	Living alone	2.37 (0.98-5.73)
45-64	Male	Low	Immigrant	LWO	2.53 (2.14-2.98)
65-84	Female	High	Immigrant	Living alone	2.58 (0.96-6.89)
30-44	Female	High	Immigrant	Living alone	2.62 (0.98-7.02)
45-64	Male	Middle	Immigrant	Living alone	2.63 (1.86-3.7)
30-44	Female	Low	Immigrant	Living alone	2.64 (1.89-3.68)
30-44	Male	Middle	Immigrant	Living alone	2.66 (1.74-4.08)
30-44	Male	Low	Immigrant	LWO	2.73 (2.32-3.2)
30-44	Male	Low	Immigrant	Living alone	2.94 (2.31-3.74)
45-64	Male	Low	Immigrant	Living alone	2.96 (2.45-3.58)
AUC					0.65
ΔAUC compared with model 6					0.01

Table 2. Prevalence ratios (PR) and 95%-confidence intervals (CI), of smoking among people aged 30-84 included in the National Health Surveys between 2004 and 2018 in relation to survey year, age, gender, income, migration- and household composition. Model 7 includes the same variables as model 6 but as a multicategorical variable, The PRs for model 7 are presented in the table 1. AUC-values with 95%CI representing the discriminatory accuracy and ΔAUC-values of the models are also presented.

		Model 1	Model 2	Model 3	Model 4	Model 5	Model 6
Year							
	2004	2.25 (2.11-2.40)	2.08 (1.95-2.21)	2.08 (1.95-2.21)	1.85 (1.74.1.98)	1.88 (1.76-2.00)	1.84 (1.73-1.97)
	2005	2.17 (2.01-2.35)	2.01 (1.86.1.17)	2.01 (1.95-2.21)	1.83 (1.69-1.98)	1.85 (1.71-2.00)	1.83 (1.69-1.98)
	2006	2.02 (1.86-2.18)	1.87 (1.72-2.02)	1.87 (1.72-2.02)	1.70 (1.57-1.84)	1.71 (1.58-1.85)	1.68 (1.55-1.82)
	2007	2.00 (1.84-2.17)	1.86 (1.71-2.01)	1.86 (1.71-2.01)	1.71 (1.57-1.85)	1.73 (1.59-1.87)	1.70 (1.56-1.84)
	2008	1.89 (1.76-2.02)	1.76 (1.64-1.88)	1.76 (1.64-1.88)	1.64 (1.53-1.75)	1.65 (1.54-1.77)	1.63 (1.52-1.74)
	2009	1.87 (1.75-2.01)	1.75 (1.64.1.88)	1.76 (1.64-1.88)	1.65 (1.54-1.77)	1.66 (1.55-1.78)	1.63 (1.52-1.75)
	2010	1.75 (1.63-1.87)	1.70 (1.58-1.82)	1.70 (1.58-1.82)	1.61 (1.50-1.72)	1.62 (1.51-1.73)	1.59 (1.48-1.70)
	2011	1.57 (1.46-1.69)	1.53 (1.43-1.64)	1.53 (1.43-1.64)	1.45 (1.35.1.56)	1.46 (1.36-1.57)	1.43 (1.33-1.54)
	2012	1.56 (1.45-1.68)	1.53 (1.42-1.64)	1.53 (1.42-1.64)	1.47 (1.37-1.57)	1.47 (1.37-1.58)	1.44 (1.34-1.55)
	2013	1.52 (1.42-1.64)	1.49 (1.39-1.60)	1.49 (1.39-1.60)	1.45 (5 (1.35.1,55)	1.45 (1.35-1.56)	1.42 (1.32-1.52)
	2014	1.46 (1.36-1.57)	1.45 (1.35-1.56)	1.45 (1.35-1.56)	1.41 (1.31-1.52)	1.42 (1.32-1.53)	1.39 (1.30-1.50)
	2015	1.42 (1.32-1.53)	1.40 (1.30-1.51)	1.40 (1.30-1.51)	1.37 (1.27-1.47)	1.38 (1.28-1.48)	1.35 (1.26-1.46)
	2016	1.07 (0.99-1.16)	1.06 (0.97-1.14)	1.06 (0.97-1.14)	1.04 (0.96-1.13)	1.05 (0.97-1.13)	1.03 (0.95-1.11)
	2018	Reference	Reference	Reference	Reference	Reference	Reference
Age							
	30-44		Reference	Reference	Reference	Reference	Reference
	45-64		1.06 (1.03-1.10)	1.06 (1.03-1.10)	0.94 (0.91-0.97)	0.94 (0.91-0.98)	0.93 (0.90-0.96)
	65-84		068 (0.65-0.71)	0.68 (1.86-0.71)	0.56 (0.53-0.58)	0.57 (0.55-0.59)	0.53 (0.51.0.56)
Gender							
	Male			Reference	Reference	Reference	Reference
	Female			1.00 (0.97-1.02)	1.02 (0.99-1.05)	1.02 (0.99-1.05)	1.01 (0.98-1.04)
Income							
	Low				1.96 (1.88-2.04)	1.96 (1.88-2.04)	1.93 (1.86-2.01)
	Middle				1.42 (1.36-1.48)	1.43 (1.37-1.45)	1.42 (1.36-1.49)
	High				Reference	Reference	Reference

For peer review only

Born in Sweden							
	Native					Reference	Reference
	Immigrant					1.39 (1.34-1.45)	1.39 (1.34-1.44)
Household composition							
	Living alone						1.53 (1.48-1.58)
	Living with other(s)						Reference
AUC		0.58 (0.58-0.59)	0.60 (0.60-0.61)	0.60 (0.60-0.61)	0.64 (0.63-0.64)	0.64 (0.64-0.64)	0.64 (0.64-0.65)
ΔAUC		-	0.02	0.00	0.04	0.00	0.01

For peer review only

For peer review only

Supplementary material 4

S4. Smoking prevalence across regions of birth among 110 044 individuals responding National Health Surveys during 2004 – 2016 and 2018. Both everyday smokers and sometimes smokers are included in the proportions presented.

Region of birth	Women	Men
Sweden	17.5%	16.4%
Nordic countries	21.7%	23.0%
Europe	24.0%	27.1%
Outside Europe	16.3%	32.5%

For peer review only

STROBE Statement for manuscript entitled "Understanding the Complexity of Socioeconomic Disparities in smoking prevalence: Combining Intersectionality theory with analysis of individual heterogeneity and discriminatory accuracy (AIHDA)"

	Item No	Recommendation	
**Title and abstract**	1	(*a*) Indicate the study's design with a commonly used term in the title or the abstract	✓ p1
		(*b*) Provide in the abstract an informative and balanced summary of what was done and what was found	✓ p2
**Introduction**			
Background/rationale	2	Explain the scientific background and rationale for the investigation being reported	✓ p5-7
Objectives	3	State specific objectives, including any prespecified hypotheses	✓ p7
**Methods**			
Study design	4	Present key elements of study design early in the paper	✓ p7-8
Setting	5	Describe the setting, locations, and relevant dates, including periods of recruitment, exposure, follow-up, and data collection	✓ p7-8
Participants	6	(*a*) Give the eligibility criteria, and the sources and methods of selection of participants	✓ p8
Variables	7	Clearly define all outcomes, exposures, predictors, potential confounders, and effect modifiers. Give diagnostic criteria, if applicable	✓ p8-9
Data sources/ measurement	8*	For each variable of interest, give sources of data and details of methods of assessment (measurement). Describe comparability of assessment methods if there is more than one group	✓ p9
Bias	9	Describe any efforts to address potential sources of bias	✓ p15-17
Study size	10	Explain how the study size was arrived at	NA
Quantitative variables	11	Explain how quantitative variables were handled in the analyses. If applicable, describe which groupings were chosen and why	✓ p8-9
Statistical methods	12	(*a*) Describe all statistical methods, including those used to control for confounding	✓p9 -11
		(*b*) Describe any methods used to examine subgroups and interactions	✓ 11
		(*c*) Explain how missing data were addressed	✓p8,15
		(*d*) If applicable, describe analytical methods taking account of sampling strategy	NA
		(*e*) Describe any sensitivity analyses	✓ 14-16
**Results**			
Participants	13*	(a) Report numbers of individuals at each stage of study—eg numbers potentially eligible, examined for eligibility, confirmed eligible, included in the study, completing follow-up, and analysed	✓ p7-8
		(b) Give reasons for non-participation at each stage	✓ p8
		(c) Consider use of a flow diagram	✓ Fig.1
Descriptive data	14*	(a) Give characteristics of study participants (eg demographic, clinical, social) and information on exposures and potential confounders	✓, S.4
		(b) Indicate number of participants with missing data for each variable of interest	NA, excluded

For peer review only - http://bmjopen.bmj.com/site/about/guidelines.xhtml

Outcome data	15*	Report numbers of outcome events or summary measures	✓ p11
Main results	16	(*a*) Give unadjusted estimates and, if applicable, confounder-adjusted estimates and their precision (eg, 95% confidence interval). Make clear which confounders were adjusted for and why they were included	✓ fig.2, tab2, S2
		(*b*) Report category boundaries when continuous variables were categorized	✓ 8
		(*c*) If relevant, consider translating estimates of relative risk into absolute risk for a meaningful time period	NA
Other analyses	17	Report other analyses done—eg analyses of subgroups and interactions, and sensitivity analyses	✓ 15-17
**Discussion**			
Key results	18	Summarise key results with reference to study objectives	✓ p13-14
Limitations	19	Discuss limitations of the study, taking into account sources of potential bias or imprecision. Discuss both direction and magnitude of any potential bias	✓ p15-16
Interpretation	20	Give a cautious overall interpretation of results considering objectives, limitations, multiplicity of analyses, results from similar studies, and other relevant evidence	✓ p13-16
Generalisability	21	Discuss the generalisability (external validity) of the study results	✓ p16
**Other information**			
Funding	22	Give the source of funding and the role of the funders for the present study and, if applicable, for the original study on which the present article is based	✓ p18-19

*Give information separately for exposed and unexposed groups.

Note: An Explanation and Elaboration article discusses each checklist item and gives methodological background and published examples of transparent reporting. The STROBE checklist is best used in conjunction with this article (freely available on the Web sites of PLoS Medicine at http://www.plosmedicine.org/, Annals of Internal Medicine at http://www.annals.org/, and Epidemiology at http://www.epidem.com/). Information on the STROBE Initiative is available at www.strobe-statement.org.

For peer review only
